# Lipoprotein(a): Just an Innocent Bystander in Arterial Hypertension?

**DOI:** 10.3390/ijms241713363

**Published:** 2023-08-29

**Authors:** Gabriele Brosolo, Andrea Da Porto, Stefano Marcante, Alessandro Picci, Filippo Capilupi, Patrizio Capilupi, Luca Bulfone, Antonio Vacca, Nicole Bertin, Cinzia Vivarelli, Jacopo Comand, Cristiana Catena, Leonardo A. Sechi

**Affiliations:** 1 Department of Medicine, University of Udine, 33100 Udine, Italy; andrea.daporto@uniud.it (A.D.P.); stefanomarcante@outlook.it (S.M.); aless.picci@gmial.com (A.P.); filippocapilupi@gmail.com (F.C.); patrizio.capilupi@gmail.com (P.C.); luca.bulfone1@gmail.com (L.B.); antonio.vacca94@gmail.com (A.V.); nicole.bertin@asufc.sanita.fvg.it (N.B.); cinzia.vivarelli@asufc.sanita.fvg.it (C.V.); jacopo.comand@spes.uniud.it (J.C.); cristiana.catena@uniud.it (C.C.); 2 European Hypertension Excellence Center, Clinica Medica, University of Udine, 33100 Udine, Italy; 3 Diabetes and Metabolism Unit, Clinica Medica, University of Udine, 33100 Udine, Italy; 4 Thrombosis and Hemostasis Unit, Clinica Medica, University of Udine, 33100 Udine, Italy

**Keywords:** lipoprotein(a), hypertension, hypertensive organ damage, cardiovascular disease, renal function, endothelial cells, vascular smooth muscle cells, arterial stiffness, antisense oligonucleotides, small interfering RNA

## Abstract

Elevated plasma lipoprotein(a) [Lp(a)] is a relatively common and highly heritable trait conferring individuals time-dependent risk of developing atherosclerotic cardiovascular disease (CVD). Following its first description, Lp(a) triggered enormous scientific interest in the late 1980s, subsequently dampened in the mid-1990s by controversial findings of some prospective studies. It was only in the last decade that a large body of evidence has provided strong arguments for a causal and independent association between elevated Lp(a) levels and CVD, causing renewed interest in this lipoprotein as an emerging risk factor with a likely contribution to cardiovascular residual risk. Accordingly, the 2022 consensus statement of the European Atherosclerosis Society has suggested inclusion of Lp(a) measurement in global risk estimation. The development of highly effective Lp(a)-lowering drugs (e.g., antisense oligonucleotides and small interfering RNA, both blocking LPA gene expression) which are still under assessment in phase 3 trials, will provide a unique opportunity to reduce “residual cardiovascular risk” in high-risk populations, including patients with arterial hypertension. The current evidence in support of a specific role of Lp(a) in hypertension is somehow controversial and this narrative review aims to overview the general mechanisms relating Lp(a) to blood pressure regulation and hypertension-related cardiovascular and renal damage.

## 1. Introduction

Lipoprotein(a) [Lp(a)] was first described by Kare Berg in 1963 [1] and its strong association with coronary artery disease (CAD) was reported in the early 1970s in many retrospective and case–control studies [2,3,4,5,6,7,8,9,10,11,12]. These observational studies, however, could not establish evidence of a causal role of Lp(a) as opposed to the possibility to be just a disease marker. Results of subsequent prospective investigations [13,14,15,16,17,18,19,20,21,22,23,24] that were conducted in the early 1990s were somewhat discordant as to the contribution of plasma Lp(a) to the development of CAD, and this is why the scientific interest in Lp(a) was dampened, dragging this lipoprotein temporarily out of the picture. During the last 15 years, publication of many landmark reports (prospective, population-based studies; meta-analyses of prospective, population-based studies; Mendelian randomization studies; genome-wide association studies) made this landscape rapidly change and Lp(a) has entered a new era of heightened interest, emerging again as a robust genetic, independent, and realistically causal risk factor for atherosclerotic cardiovascular disease (CVD) and calcific aortic valve disease [25,26,27,28,29,30,31,32,33,34,35,36,37,38,39,40,41,42,43,44,45,46,47]. In the setting of primary prevention, measurement of Lp(a) can reclassify up to 40% of patients at intermediate risk of CVD (according to Framingham and Reynolds risk scores) either to a higher or a lower risk category [48]. In secondary prevention, evidence coming from the recent AIM-HIGH [49], JUPITER [50], and LIPID trials [51] underscore the concept that elevated Lp(a) increases the risk of recurrent cardiovascular events despite optimal LDL-C reduction on statin therapy. These studies support the hypothesis that genetically elevated Lp(a) plays a pivotal role in determining “residual cardiovascular risk” and provide a rationale to develop and test specific Lp(a) lowering agents. Not surprisingly, in line with the aforementioned bulk of evidence, many recent national and international guidelines and consensus statements have been published on Lp(a) testing and treatment [52,53,54,55,56]. All these documents do recognize elevated Lp(a) as a critical factor for reclassification of cardiovascular risk and recommend measurement of Lp(a) in individuals at intermediate-to-high risk and subjects with a family history of premature CVD.

From a historical perspective, Lp(a) has been the least studied of the four main clinical categories of lipid disorders (elevated LDL-C, low-HDL-C, elevated triglycerides, and elevated Lp[a]), being the interest of lipidologists primarily focused on LDL-C because of its predominant role in CVD and important benefits of LDL-C-lowering treatments in prevention. Conversely, current lipid-lowering treatments and dietary changes have yielded only unremarkable effects on Lp(a) levels, and potential cardiovascular benefits of Lp(a) reduction are all yet to be proven [57]. The recent development of specific Lp(a)-lowering agents [58,59] has paved the way towards the possibility to test the hypothesis that Lp(a) reduction decreases the risk of CVD. However, while awaiting the results of the ongoing phase-3 Lp(a)-HORIZON trial (NCT04023552) involving small interfering RNA inhibition of hepatic synthesis of Lp(a) and, as indicated by current guidelines, this heterogenous lipoprotein cannot be considered a target of treatment. Furthermore, the growing interest in targeting plasma Lp(a) levels is considerably hampered by significant gaps in the knowledge of its metabolism and pathophysiological mechanisms [60]. At least in part, these gaps can be attributed to the lack of physiologically adequate animal models and large part of the mechanistic evidence on Lp(a) contribution to atherothrombotic events comes from in vitro studies [61].

Arterial hypertension is the most common chronic disease worldwide [62,63], with a prevalence that exceeds 30% in many adult populations, accounting for approximately 10 million yearly deaths [64]. For these reasons, hypertension is broadly considered the leading modifiable cardiovascular risk factor, increasing the risk of morbidity, disability, and mortality related to CVD [65]. Although the propensity of hypertensive patients to develop cardiovascular and renal damage is directly related to the direct influence of high blood pressure (BP), additional factors, including circulating lipids, play an important role. In fact, dyslipidemia is often detected in hypertensive patients, and circulating levels of specific lipoproteins, including Lp(a), can contribute to the development of subclinical and overt hypertension-related organ damage [66,67].

The aim of this narrative review is to update the current views on the specific involvement that Lp(a) might have in BP regulation and, most importantly, in the development of hypertension-related organ damage. We systematically searched the medical literature published in the English language using the Pubmed MeSH and the key terms “blood pressure”, “arterial hypertension”, “hypertensive-organ damage”, “lipoprotein(a)”, and “apolipoprotein(a)”, for extraction. We considered only full-text articles with original experimental animal or human data and systematic reviews and meta-analyses reporting on the effects of Lp(a) on mechanisms of BP regulation and hypertension-related cardiac, vascular, and renal subclinical and clinical damage. Articles were retrieved by G.B. and were independently reviewed by C.C. and L.A.S. and subsequently discussed together for final article selection. Article selection was performed according to the quality of evidence of studies that was defined following the Grading of Recommendations, Assessment, Development, and Evaluations (GRADE) criteria that are based upon the study design, dimension, consistency, and magnitude and dose-dependency of effect [68]. Only studies rated with moderate-to-high GRADE ratings of certainty were considered.

## 2. Lipoprotein(a) Structure, Genetics, Metabolism, Distribution of Plasma Concentration Levels, and Measurement Methods

### 2.1. Lp(a) Structure

**Figure 1:**
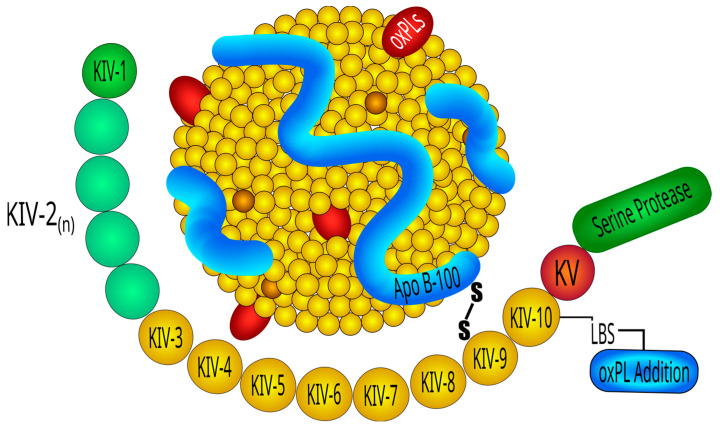
Structure of lipoprotein(a). Lipoprotein(a) consists of an LDL-like core lipoprotein molecule, containing apolipoprotein-B100 to which a glycoprotein of variable molecular weight, apolipoprotein (a) [apo(a)] comprising multiple heterogeneous triple-loop structures called kringles, is covalently linked via a single cysteine–cysteine disulfide bond. K IV, kringle 4; LBS, lysine binding sites; oxPL, oxidized phospholipids.

Lp(a) consists of an LDL-like core lipoprotein molecule, containing apolipoprotein B (apo-B), to which a glycoprotein of variable molecular weight, apolipoprotein (a) [apo(a)], is covalently linked via a single cysteine–cysteine disulfide bond [69] (Figure 1). Lp(a) particles contain apo(a) and apo-B100 in a 1-to-1 molar ratio [70]. The lipoprotein moiety is essentially indistinguishable from LDL regarding its physical chemical properties and consists of a hydrophobic core of esterified cholesterol and triglicerides, surrounded by a surface monolayer of phospholipids, unesterified cholesterol, and other proteins [71]. The peculiar characteristics and the size variability of Lp(a) that is the main determinant of its plasma concentration are almost entirely accounted for by the presence of apo(a). Apo(a) is encoded by the LPA gene, located on chromosome 6q26 [72], and cloning of a c-DNA encoding apo(a) revealed a high degree of homology of this lipoprotein with the fibrinolytic proenzyme plasminogen [73]. Both molecules contain coding sequences forming multiple triple-loop structures called kringles (K) [74] that, due to resemblance of shape, were named after Danish pastries [75]. The characteristic tri-looped arrangement of the kringle structure is stabilized by the presence of three internal disulfide bridges, resulting from the interaction of six conserved cysteine residues [74]. Plasminogen contains an N-terminal tail domain that is attached to one copy each of five kringles, designated as kringle-1 through kringle-5, and a trypsin-like protease domain [76]. In contrast to plasminogen, apo(a) lacks the tail domain and the first three kringle domains of plasminogen and instead is formed of multiple repeated copies of sequences homologous to plasminogen kringle-4 (K4) domain, followed by a single kringle-5-like domain and an inactive protease-like domain [77]. Lp(a) contains 10 subtypes of K4 repeats (K4 type-1 to K4 type-10) that differ from each other based on aminoacidic sequence. All K4 kringle types are present in a single copy within the Lp(a) moiety, with the notable exception of K4 type-2, which is present in a variable number of identically repeated copies, usually ranging from 3 to more than 40 [70], that are encoded by the LPA gene. This important variation leads to the size heterogeneity in apo(a) isoforms found in the general population. As a rule, apo(a) isoform size is inversely related to plasma Lp(a) concentration in most populations [78]. Kringles are ligand-binding sites and as such serve critical functions and pathobiological roles that are mediated by their lysine-binding sites (LBS). K4 type-9 forms a covalent disulfide bridge to the apo-B100 moiety of LDL and is critical in the creation of the covalent apo(a) LDL-complex whose formation is crucially initiated by noncovalent interaction between LBS of apo(a) and lysine residues of apoB100. The lysine binding site in K4 type-10 is thought to mediate the binding of Lp(a) to different substrates including fibrin, cell surface receptors, and extracellular matrix proteins [79,80,81,82]. Moreover, in recent years, K4 type-10 has emerged as a key component for the pathogenic potential of Lp(a) as it contains the site to which oxidized phospholipids (OxPL), are covalently bound to two histidine residues [83,84,85]. Kringles have also been identified in several other proteases involved in the coagulation process and fibrinolysis, including prothrombin, factor XII, tissue-type plasminogen activator, and urokinase-type plasminogen activator [86]. In addition to apoB-100 and apo(a), which account for 88% of the total protein mass of Lp(a), recent proteomic analysis has shown a more complex composition of Lp(a) [87,88], with more than 35 proteins identified on its surface.

### 2.2. Lp(a) Genetics

The apo(a) gene (LPA) is located on chromosome 6q26-q27 in linkage with the plasminogen gene [72] and is highly expressed in the liver [89,90] but not in other organs [73]. The apo(a) gene is composed of four main regions: the sequence coding for the signal peptide, the sequence coding for the plasminogen-like 4 domains (K4) containing several tandem repeats of a 5.5 kB sequence encoding a cysteine-rich motif, the sequence for the plasminogen-like 5 domain (K5), and the sequence coding for the plasminogen-like protease domain [73]. The extensive structural homology of the apo(a) gene with the plasminogen gene (78–100%) [73] and the proximity of the two genes suggest a common origin, with apo(a) gene diverging from the plasminogen gene during primate evolution about 40 million years ago, and evolving over time through duplications, deletions, gene conversions, and point mutations [91]. The plasminogen gene consists of five different kringle domains (K1 to K5), each present as single copies [72,73]; in the Lp(a) gene K1 to K3 were lost by deletion, whereas K4 expanded and differentiated into 10 different types of K4 domains, each with specific aminoacidic composition. While K4 type-1 and K4 type-3 to K4 type-10 are present only as single copies [92], the K4 type-2 further replicated, resulting, as previously stated, in multiple copies (2 to >40 repeats, with a repeat size of 5.5 kB) [93]. This accounts for the extensive size heterogeneity in the apo(a) gene and consequently in the apo(a) protein because the K4 type-2 copy number variation, also termed “apo(a) size polymorphism”, is the major determinant of Lp(a) isoform size. Therefore, the number of K4 type-2 encoding sequences results in more than 40 isoforms and more than 40 different sizes of Lp(a) particle, with substantial molecular mass variation (200–800 kilodaltons) [58,74]. Within the general population, homozygosity for the apo(a) size is rare and more than 80% of individuals carry two different-sized apo(a) isoforms, each inherited from one parent [74]. However, it should be noted that 20% of subjects express as protein only one isoform, although two isoforms are transcribed at the DNA level.

As previously stated, a strong inverse association exists between the number of K4 type-2 copies and the circulating level of Lp(a) [75,94]. Individuals with small apo(a) isoforms (≤22 K4 type-2 repeats) have higher Lp(a) concentrations and 2–4-fold higher risk of CVD [95,96], whereas those carrying large apo(a) isoforms (>22 K4 type-2 repeats) have lower Lp(a) levels and no increase in risk of CVD [97,98]. This inverse relationship between isoform size and Lp(a) concentration might be explained by a slower intracellular processing, assembling, and secretory rate of larger Lp(a) molecules [99,100,101].

Expression of LPA is regulated both at the transcriptional and post-transcriptional levels, although considerable controversy exists concerning the regulatory factors. Studies in humans have suggested that acute-phase inducers increase and sex hormones decrease LPA expression and apo(a) levels, but results are highly controversial [102]. Functional analyses of LPA conducted in transgenic mice expressing apo(a) identified two candidate regions that possess gene enhancer activities and are accessible to nuclear transcription factors [103]. One of these regions is located 26 kb away from the apo(a) gene promoter and its activity is blocked by estrogens [104]. The other region is called apo(a) transcription control region and exhibits the strongest stimulating activity over the apo(a) promoter, thereby contributing to LPA expression in vivo [105]. Characterization of the apo(a) gene promoter in HepG2 cell lines identified two composite regulatory regions, one located proximally that activates apo(a) gene transcription and one located distally that represses transcription [106]. Influence of endogenous and exogenous factors on these complex mechanisms that regulate LPA expression was reported [107,108,109], although most of these studies require confirmation. Thus, more research will be needed to better clarify the mechanisms that contribute to transcriptional and post-transcriptional regulation of apo(a) gene expression.

In addition to LPA gene expression, other genetic determinants of Lp(a) concentration have been identified, including pentanucleotide repeats in the promoter region (PNRP) [110,111], variants that affect RNA splicing, and single nucleotide polymorphisms (SNPs) within the structural and functional domains [112,113,114,115]. More than 200 single nucleotide polymorphisms (SNPs) have been identified by genome-wide association studies [116,117] showing variable influence on Lp(a) levels. Some of these SNPs are associated with elevated Lp(a) levels, while others are associated with low Lp(a) levels. However, not all variants are demonstrated to be relevant in predicting Lp(a) levels [118,119,120,121,122]. In these studies, only rs3798220, located in the protease-like domain of apo(a) and rs10455872, which maps to intron 25, have repeatedly been associated with an increased Lp(a) level and a reduced copy number of K4 repeats.

In summary, Lp(a) is highly heritable with plasma concentration predominantly determined by variation in the LPA gene (accounting for >70–90% of variance), and is not significantly influenced by age, sex, physical activity, and diet. The K4 type-2 copy number variation which determines the apo(a) isoform size, explains from 20% to 77% of the variation in Lp(a) concentration depending on the population and methods used, whereas taken together, specific clusters of SNPs in various regions of the LPA gene account for 35–40% of variability.

### 2.3. Lp(a) Metabolism

Lp(a) biosynthesis faces four main steps: transcription of LPA, protein translation, transfer to the secretory pathway, and assembly of the Lp(a) particles. Lp(a) is exclusively produced in the liver which secretes apo(a)- and apoB-containing lipoproteins separately, so that the final assembly of Lp(a) takes place extracellularly by covalent linkage of apo(a) with apoB [123]. Synthesis and secretion are regulated by the effects of genetic control of LPA expression and processing of the apo(a) protein, respectively. Recognition that LPA expression is closely linked to Lp(a) levels established the premises for the development of antisense oligonucleotides to prevent apo(a) translation and lower circulating Lp(a) [124].

Catabolism of Lp(a) is not entirely clear. Regulation and function of the endocytic receptor which removes Lp(a) from the circulation is still a matter of debate [125]. The presence of apo(a) and perhaps also involvement of proprotein convertase subtilisin/kexin type 9 (PCSK9) limits removal of Lp(a) by the LDL-receptor. In addition, several other endocytic receptors have been implicated to mediate removal of Lp(a) from blood, including LDL-receptor related protein 1, very low density lipoprotein receptor, scavenger receptor B1, and plasminogen receptor KT (PlgRKT) [125,126]. While most lipoprotein receptors direct Lp(a) into a route which leads to the lysosomal degradation of the entire particle, PlgRKT was reported to shuttle Lp(a) into a pathway which leads to the selective degradation of the lipids and apoB, but to the re-secretion of apo(a) which then associates with another LDL-particle to form a new Lp(a) particle.

### 2.4. Distribution of Lp(a) Concentration and Effects of Non-Genetic Factors

The distribution of plasma Lp(a) levels is highly variable among different ethnic groups with concentrations varying up to 1000-fold within each population, ranging from less than 0.1 mg/dL to as high as 387 mg/dL. The lowest levels are seen in non-Hispanic Caucasians, Chinese, and Japanese; slightly higher levels have been documented in Hispanics, while the highest levels are found in Blacks [127]. In Caucasians, plasma levels are comparable in men and women, and it is estimated that 20% of the population worldwide has an Lp(a) level >50 mg/dL (>105 nmol/L) [128], 5% of individuals has an Lp(a) level above 120 mg/dL (250 nmol/L), whereas only 1% of individuals has an extremely elevated Lp(a) level above the 99th percentile, corresponding approximately to 180 mg/dL. Plasma levels are generally unaffected by dietary interventions or various physiological and environmental factors, including age, sex, fasting state, or physical activity, but are also known to be slightly influenced by pregnancy, menopause, hormone use, cholestasis, thyroid dysfunction, acute phase events, and renal function [129]. Elevated Lp(a) levels have been observed in patients with chronic kidney disease, being inversely correlated with glomerular filtration rate, with an increase that is independent on the apo(a) phenotype, strongly suggesting a leading role of the kidney in Lp(a) removal from blood [130].

### 2.5. Lp(a) Measurement

Reproducible and reliable measurement of Lp(a) was difficult to obtain mainly because of the highly polymorphic nature of the apo(a) moiety, due to the variation in isoform size. Additional factors included lack of standardization across laboratories with some assays reporting Lp(a) values as mass concentrations (mg/dL) and others as particle concentrations (nmol/L) [131], and the adoption of antibody-based approaches which led to possible underestimation of the small isoforms and overestimation of the large isoforms [55]. The efforts of The International Federation of Clinical Chemistry and Laboratory Medicine to standardize reference material to calibrate Lp(a) assays improved the reproducibility between methods [132], although some degree of heterogeneity might persist [133]. The recently developed LC–MS/MS method which recurs to selected proteotypic peptides quantified by isotope dilution is isoform-independent, overcoming some of the limitations and allowing better standardization of assays [134].

## 3. Plasma Lp(a) Concentrations in Hypertension

Dyslipidemia is more prevalent in hypertensive than normotensive individuals, and changes in lipid levels progressively worsen with increasing BP [135]. Increased levels of total and low-density lipoprotein cholesterol and triglycerides, and lower high-density lipoproteins cholesterol were reported in hypertensive patients [136]. Regarding Lp(a), data are highly controversial mostly depending upon lack of standardization of assays and relevant differences among ethnic groups. While some studies reported higher Lp(a) concentrations in hypertensive than normotensive subjects, other studies did not [65,137,138,139,140,141,142,143,144,145,146]. In a study conducted by Lip et al. on ambulatory hypertensive patients, median Lp(a) levels were found to be markedly elevated in Blacks, in line with previous observations [144]. Elevated plasma Lp(a) was also more frequent in hypertensive patients of Indian than Caucasian descent, although in hypertensive Caucasians no differences were observed with the respective normotensive subjects. In agreement with these findings, two additional studies reported an increased prevalence of elevated Lp(a) in Indian hypertensives free of cardiovascular complications in comparison to their respective normotensive controls [147,148]. Moreover, a significantly growing prevalence of elevated plasma Lp(a) levels was demonstrated with increasing severity of hypertension (51%, 55%, and 91.67% in grade 1, 2, and 3 of hypertension, respectively) in Indian patients [148]. This association was not found in any other ethnic group, although in an Australian cohort of hypertensive patients, a greater prevalence of plasma Lp(a) > 50 mg/dL was associated with use of a higher number of antihypertensive drugs [145]. Thus, differences in Lp(a) levels between subjects with normal or increased BP appears to be limited to some selected ethnic groups. This might be related to their different genetic background affecting LPA gene expression, together with possible epigenetic influences due to differences in environmental components primarily related to dietary habits.

## 4. Lp(a) and The Vascular Wall

Essential hypertension is the most frequent form of hypertension and is characterized by a complex and multifactorial pathophysiology, where blood vessels, heart, and kidneys are reciprocally involved in regulation of the leading determinants of systemic BP, namely cardiac output and peripheral vascular resistance [149]. Within this complex interplay, a crucial role belongs to vascular endothelium that, in normal conditions, balances vasoconstriction and vasodilation of resistance vessels, also exerting important antithrombotic and anti-inflammatory functions that might be impaired in the process of atherogenesis [150]. A dysfunctional endothelium has been extensively demonstrated in hypertension [151], and a large body of evidence suggests that high BP and endothelial dysfunction influence each other, giving raise to a pathogenetic vicious circle [152]. Endothelial dysfunction can be defined as a phenotypic modification that is characterized by a shift of its actions toward reduced vasodilation, vascular cell proliferation, platelet adhesion, and activation and proinflammatory and prothrombic mechanisms [153].

**Figure 2:**
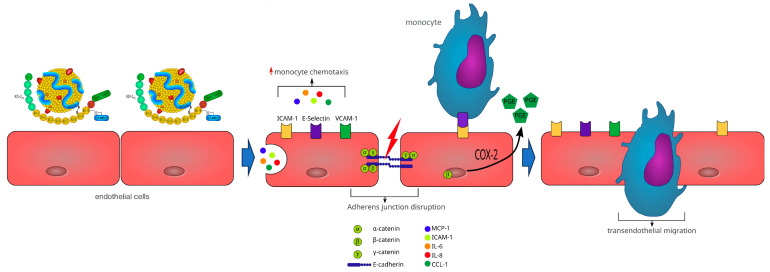
Consequences of lipoprotein(a) interaction with vascular endothelium. Lp(a) crosses endothelial surfaces bound to oxidized phospholipids and binds to subendothelial structures. Once retained and oxidized, Lp(a) modifies the properties of endothelial cells that enhance expression of cell adhesion molecules and monocyte chemotaxis. Enhanced contraction and loss of contact of endothelial cells due to increased phosphorylation of myosin light chains and rearrangement of the actin cytoskeleton disrupts the integrity of the endothelial monolayer, leading to increased permeability and transendothelial migration of monocytes. Moreover, migration and adhesion of progenitor endothelial cells is impaired together with activation of endothelial cells apoptosis. K IV, kringle IV; LBS, lysine binding sites; oxPL, oxidized phospholipids; ICAM-1, intercellular adhesion molecule-1; VCAM-1, vascular cell adhesion molecule-1; COX-2, cyclooxygenase-2; PGE, prostaglandin E; MCP-1, monocyte chemoattractant protein-1; IL-6, interleukin-6; IL-8, interleukin-8; CCL-1, CC chemokine ligand-1.

In vitro studies indicate that elevated Lp(a) can directly contribute to atherogenesis and cause endothelial cell (ECs) and vascular smooth muscle cell (VSMCs) dysfunction (Table 1). These effects appear to be prevalently mediated by the apo(a) moiety, due to its hydrophilic properties which allow a direct interaction with the vascular endothelium as well as other cellular receptors [154]. Much like other lipoproteins, Lp(a) can diffuse passively through endothelial surfaces via concentration gradients, accumulating in subendothelial spaces where, after binding to proteoglycans and other subendothelial structures, becomes oxidized, promotes inflammation, and mediates atherogenesis [155]. Lp(a) accumulation and retention in the vessel wall and sub-endothelial surfaces is facilitated by a potent lysine-binding pocket present on K4 type-10 that binds to exposed lysine on denuded endothelial surfaces and to components of the subendothelial matrix [156]. Moreover, Lp(a) is the preferential lipoprotein carrier of oxidized phospholipids (OxPLs), markedly increasing its proinflammatory properties in comparison to other atherogenic lipoproteins [81,82,157,158]. Once retained and oxidized, Lp(a) modifies the properties of endothelial cells (ECs) that are shifted towards a more inflammatory phenotype characterized by: (a) enhanced expression of cell adhesion molecules (VCAM-1, E-selectin, and ICAM-1) [159,160]; (b) increased oxidative stress with increased generation of reactive oxygen species leading to accelerated senescence [161] and disruption of the integrity of ECs, leading in turn to increased permeability of the endothelial monolayer [162]; (c) enhanced contraction and loss of contact of ECs due to increased phosphorylation of myosin light chains and rearrangement of actin cytoskeleton through the Rho/Rho-kinase-dependent signaling pathway [163,164]; (d) impaired adhesion and migration of endothelial progenitor cells (EPCs) [165]; (e) impairment of angiogenesis signaling pathways and enhanced ECs apoptosis [166] (Figure 2).

In animal models, high Lp(a) levels impair endothelium-dependent vasodilation, as demonstrated by a dose-dependent reduction in the expression of inducible nitric oxide synthase, both at mRNA and protein level [167,168]. Lp(a) can also affect vascular smooth muscle cells (VSMCs), as suggested by early in vitro studies showing that cell migration and proliferation could be triggered by apo(a)-mediated downregulation of plasmin-dependent activation of transforming growth factor-β [169]. Moreover, apo(a) interacts with integrin αVβ3 on the surface of VSMCs and signals through tyrosine kinases to activate RhoA, thereby causing stress fiber formation, cell spreading, and chemorepulsion, with a negative impact on VSMCs remodeling and progression of atherosclerosis and arterial stiffening [170].

Despite the large body of experimental data, evidence obtained in vivo on Lp(a)-mediated effects on functional and structural vascular changes is limited to a few small studies. Early studies reported that elevated Lp(a) concentration is related to endothelial dysfunction in children with familial hypercholesterolemia [171], healthy adolescents [172], and healthy adults [173]. In patients with angiographically normal coronary arteries, elevated plasma Lp(a) levels were found to be associated with impaired coronary vasomotion induced by acetylcholine infusion [174,175], but not by dipyridamole [176]. The effects of Lp(a) on vascular response were investigated in the forearm arterial bed of healthy subjects by intraarterial infusion of increasing doses of acetylcholine, sodium nitroprusside, and N-monomethyl L-arginine (L-NMMA) [173]. Both endothelium-dependent and endothelium-independent vasodilatory responses were comparable in the groups with low, medium, and high plasma Lp(a) concentrations, whereas the endothelium-dependent vasoconstrictive response to L-NMMA infusion was greater in patients with elevated plasma Lp(a). The influence of Lp(a) levels on early vascular changes was evaluated in another study on healthy subjects in which flow-mediated (endothelium-dependent) and nitrate-mediated (smooth muscle-dependent) arterial dilations were measured by high-resolution ultrasound reporting no significant relationships with Lp(a) concentration [177]. By contrast, more recent studies that have examined in a multiethnic cohort of healthy subjects the brachial artery response by ultrasound imaging have found an inverse correlation of Lp(a) levels and low-molecular weight apo(a) with endothelium-dependent flow-mediated vasodilation, but not endothelium-independent nitrate-induced vasodilation [178]. Thus, while substantial in vitro evidence indicates that Lp(a) can impair ECs and VSMCs function in the arterial wall, the evidence obtained in vivo is controversial and still under debate.

## 5. Lp(a) and Organ Damage in Hypertension

The development of organ damage in essential hypertensive patients is likely related, but not limited to, the direct effects of increased BP levels. Additional factors, including circulating lipids, play a crucial role in the process of vascular damage since dyslipidemia, which is frequently detected in hypertension, and serum levels of specific lipoproteins greatly affect cardiovascular morbidity and mortality in hypertensive populations [179]. Retrospective and prospective studies have shown that high serum levels of Lp(a) are an independent risk factor for cardiovascular diseases [180]. Moreover, previous studies performed in large cohorts of hypertensive patients have demonstrated that serum Lp(a) levels predict the presence and severity of hypertensive organ damage [65]. In 277 untreated patients with mild-to-moderate essential hypertension and 102 matched healthy controls, we measured plasma Lp(a) and characterized apo(a) phenotypes. In hypertensive patients, organ damage was defined as stage 0 (no organ damage), stage I (subclinical organ damage), and stage II (clinically relevant organ damage) according to current guidelines, after extensive investigation of cerebral, cardiac, vascular, and renal damage. Although plasma Lp(a) was comparable in hypertensive patients and normotensive controls, Lp(a) levels increased significantly and progressively across stages of hypertension-related organ damage. In a multivariate analysis, Lp(a) levels were the best discriminator of the presence of organ damage and an increase in its concentration was associated with significantly greater expression of low molecular weight apo(a) phenotypes, suggesting a genetic predisposition to hypertensive complications linked to circulating Lp(a).

The longitudinal relationship of Lp(a) and hypertension to cardiovascular outcomes in primary prevention has been recently assessed in a retrospective analysis of the Multi-Ethnic Study of Atherosclerosis (MESA), an ongoing community-based cohort study [181]. Analysis included 6674 individuals free of clinical CVD at baseline, who were divided into four groups according to Lp(a) status (<50 mg/dL vs. >50 mg/dL) and hypertension status (hypertension vs. no hypertension). After an average follow-up of 13.9 years, CVD-free survival was comparable in normotensive subjects with low or high plasma Lp(a), whereas among subjects with hypertension, those with Lp(a) ≥ 50 mg/dL had a significantly higher risk of CVD than those with Lp(a) < 50 mg/dL (HR, 1.24; C.I., 1.01–1.53). Notably, the interaction between hypertension with Lp(a) concentrations had stronger association with increased CVD risk among Blacks (HR, 1.48; C.I., 1.09–2.02) than in other ethnic groups. In this study, although the major contribution to cardiovascular risk could be attributed to hypertension, elevated plasma Lp(a) significantly worsened the association of hypertension with cardiovascular disease. These findings suggest that high Lp(a) levels might also contribute to the residual cardiovascular risk of well-treated hypertensive patients by increasing the burden of subclinical organ damage via its well-known proatherogenic, proinflammatory, and prothrombotic effects [182,183], and interacting with the renin–angiotensin system [184].

Arterial vessels are one of the main targets of hypertension and a broad interest has gathered around the mechanisms that might contribute to arterial stiffening. Arterial stiffness is recognized as a strong predictor of cardiovascular events, both in the general population [185,186] and in hypertensive patients [187,188]. Currently, arterial stiffness can be estimated by noninvasive methods, including measurements of the augmentation index (AIx) by pulse wave analysis and carotid–femoral pulse wave velocity (PWV), which provide a comprehensive assessment of the stiffness of the entire arterial tree (elastic plus muscular arteries and arterioles) [189]. These are now widely accepted tools for the assessment of subclinical arterial damage in hypertension [61]. Increased arterial stiffness has been associated with major cardiovascular risk factors, including being overweight or obese, impaired glucose tolerance, dyslipidemia, and smoking [190,191,192,193] and is characterized by both structural and functional changes of the vascular wall [194]. Arterial stiffening results from extensive anatomical rearrangement with the depletion and fragmentation of elastin fibers and the deposition of collagen elements in the extracellular matrix. This is closely related to aging, but other factors, in addition to high blood pressure, might play a role, including endothelial dysfunction, activation of tissue-specific renin–angiotensin–aldosterone system, increased production of proinflammatory cytokines, and a prothrombotic state [194,195,196,197].

The hypothesis of a possible contribution of Lp(a) to arterial stiffening was initially investigated in elderly Japanese subjects with type 2 diabetes, reporting an independent association of its levels with the PWV [198]. A significant correlation between the plasma levels of oxidized Lp(a) and PWV was also reported in a subset of relatively old (mean age: 66 years) hypertensive patients with coronary artery disease and diabetes [199]. Similarly, in a small study enrolling hypertensive women, a significant relationship was reported between oxidized Lp(a) levels and the cardio–ankle vascular index [200]. More recently, in 138 patients with hypertension who were younger (mean age: 51 years) and free of diabetes and cardiovascular and renal complications, we measured plasma lipids and assessed AIx and PWV [66]. Plasma Lp(a) concentrations were significantly and directly related with both AIx and PWV, although multiple regression analysis showed independence of correlation of Lp(a) only with the AIx. Due to the relevance of AIx to the assessment of the peripheral arterial tree, these findings might indicate greater and more specific relevance of Lp(a) to the peripheral component of arterial stiffness.

All these findings might have considerable clinical implications for the identification of target organ damage in hypertensive patients as well as for their prevention and treatment. Lp(a) measurement might indeed be useful in the diagnostic workup of these patients, to identify those who might be more prone to developing organ damage. On the other hand, in consideration of the encouraging data coming from clinical trials of new Lp(a)-lowering therapies, in the near future, a reduction in Lp(a) levels might possibly improve hypertension outcomes. Nowadays, detection of elevated Lp(a) levels in hypertensive patients could be an alarm light and induce clinicians to pursue better control over blood pressure and additional risk factors to prevent cardiovascular events.

## 6. Lp(a) and Hypertensive Renal Damage

Despite robust evidence suggesting an inverse relationship between renal function and plasma Lp(a) levels in patients with severely impaired glomerular filtration rate [16,201], only a few studies have investigated this relationship in patients with hypertensive nephrosclerosis. Data obtained from large cohorts of subjects with end-stage renal disease that included mostly patients with diabetic nephropathy suggested a reciprocal relationship between Lp(a) levels and renal function. As stated above, an impaired kidney function affects Lp(a) catabolism, increasing its circulating levels [130] but, on the other hand, Lp(a) itself might have a role in causing renal disease progression [16,201,202]. Support to a possible role of Lp(a) in the development and progression of renal dysfunction has been provided also in other studies [203,204], although in the Chronic Renal Insufficiency Cohort (CRIC) study that enrolled 3939 adults with chronic kidney disease, mostly diabetics, none of the circulating lipids, including Lp(a), were significantly associated with progression of kidney disease [205]. Further work is needed to better understand the complex relationship between Lp(a) and renal disease, although it should be kept in mind that increased Lp(a) levels are one the main contributors to the increased cardiovascular risk found in patients with progressive renal disease [206].

In a cross-sectional study of 417 hypertensive patients, 160 of whom had glomerular filtration rate from 30 to 89 mL/min/1.73 m^2^ of body surface area, we measured serum lipids and apolipoproteins [130]. Lp(a) levels were significantly higher in patients with early impairment of renal function caused by hypertensive nephrosclerosis and, most importantly, there was a highly significant inverse relationship between Lp(a) levels and glomerular filtration rate. In these patients, elevated serum Lp(a) was also associated with greater prevalence of cerebrovascular, coronary artery, and peripheral vascular disease [207]. The inverse relationship of Lp(a) with glomerular filtration was confirmed in another group of 250 hypertensive subjects with hypertensive nephrosclerosis and early reduction of glomerular filtration in whom apo(a) phenotypes were characterized [208]. In this study, no relationship was found between serum Lp(a) and urinary protein losses and the frequency of low-molecular weight phenotypes was comparable across levels of glomerular filtration, suggesting that renal failure per sè or other genes beside LPA could be responsible for Lp(a) increase in subjects with early impairment of renal function. Finally, a contribution of Lp(a) has been demonstrated in 50 patients with angiographic evidence of atherosclerotic renal artery stenosis [209]. In these patients, multivariate analysis showed that Lp(a) was associated with renal artery stenosis independently of other confounders including renal function and history of CVD. Thus, substantial evidence suggests that in addition to the referred evidence of the impact that elevated circulating Lp(a) has on cardiovascular outcomes in hypertensive patients, a close relationship exists between this lipoprotein and hypertensive nephrosclerosis, starting from the earliest stages of renal disease.

## 7. Lipoprotein(a): Dietary and Pharmacological Interventions

Regarding lifestyle interventions, there is a historical assumption according to which diet has no effect on Lp(a). However, since the first report of dietary effects on Lp(a) in 1991 [210], there have been only a few well controlled clinical studies investigating the consequences of dietary modification on its levels. Overall, current evidence, albeit limited, indicates that diet modulates only modestly Lp(a) and often in the opposite direction to LDL-C [211,212,213,214,215,216,217,218,219]. Response of Lp(a) levels to dietary modifications is highly heterogeneous, although a trend towards increasing levels with diets rich in saturated fatty acids intake and reduction when these fatty acids are replaced by long-chain carbohydrates and unsaturated fatty acids has been reported. In hypertension, administration for 1 month of 4 g/day of polyunsaturated fatty acids to patients with plasma Lp(a) > 25 mg/dl significantly decreased these levels by 38%, whereas administration for the same time of 1 mg/day reduced Lp(a) by 17% [220]. It is our opinion that, whatever could be the effect of diet on plasma Lp(a), clinicians should keep recommending patients to replace saturated fatty acids with unsaturated fat from their diets to reduce CVD risk.

The resurgence of Lp(a) in the context of cardiovascular prevention and treatment is mainly due to the relatively recent development of new RNA-directed treatments. Indeed, traditional lipid-lowering agents had demonstrated little and clinically irrelevant effects on Lp(a). These agents included statins; niacin (20% reduction of Lp(a) at maximal dose but with an unknown effectiveness in reducing major atherosclerotic cardiovascular events-MACE) [221]; cholesteryl ester transfer protein (20–30% reduction of Lp(a) with no reduction in MACE) [222]; and apolipoprotein B100 antisense oligonucleotide mipomersen (20–40% reduction in Lp(a) levels but MACE reduction unknown in patients with elevated Lp(a) with significant concerns regarding potential hepatotoxicity which justifies limited approval for homozygous familiar hypercholesterolemia) [223]. Encouraging data have emerged for PCSK9 inhibitors (evolocumab and alirocumab) in secondary analyses of the FOURIER and ODYSSEY outcome trials [224,225], where a modest reduction of Lp(a) (27% with evolocumab, 23% with alirocumab) was independently associated with an absolute reduction in MACE (ARR 2.5% with NNT of 45 for evolocumab; ARR 2.3% with NNT 43 for alirocumab), suggesting that even a modest reduction of Lp(a) levels may translate into a significant benefit.

The novel RNA-directed therapies that specifically target the LPA gene drew roots in a study that Frank et al. published more than 20 years ago [226]. In elegant experiments performed in transgenic mice expressing apo(a), these authors provided the first demonstration that production of this apolipoprotein could be blocked by use of an apo(a)-antisense-RNA. Following this evidence, clinical applications of LPA-targeting treatments for use in humans have recently been developed. These include a single-stranded antisense oligonucleotides (ASO), which prevent gene expression through degradation of the target mRNA by RNaseH1, and a small interfering RNA (siRNA), composed of two strands (passenger strand and guide strand), with the guide strand binding to target mRNA within an RNA-induced silencing complex (RISC). These compounds have been named pelacarsen (ASO), olpasiran (siRNA), and SLN360 (siRNA), and all have remarkably and dose-dependently lowered Lp(a) levels, while maintaining a good safety profile in phase 2 and phase 1 trials (reduction of Lp(a) levels of 35–80%, 80–94%, and 46–98% within 112, 113, and 150 days by pelacarsen, olpasiran, and SLN360, respectively) [227,228,229]. The HORIZON trial (Assessing the Impact of Lipoprotein (a) Lowering With Pelacarsen [TQJ230] on Major Cardiovascular Events in Patients With CVD) (NCT 04023552), a large ongoing phase 3 multicenter, international cardiovascular outcome study with a planned duration of 4.25 years enrolling approximately 8000 patients, will provide a more definite answer to the possible contribution of pelacarsen to MACE reduction. Thus, while awaiting the results of ongoing and future outcome trials, the therapeutic strategy in patients with high Lp(a) levels should focus on reducing residual “lipid-driven” cardiovascular risk by further lowering of LDL-C levels and better control of the other cardiovascular risk factors, including hypertension.

## 8. Conclusions

The relationship between elevated Lp(a) levels and essential hypertension is still a matter of intriguing debate due to limited clinical evidence in support of a causal and/or reciprocal association. Nevertheless, solid evidence indicates that elevated Lp(a) levels can significantly contribute to cardiovascular and renal damage in hypertensive patients, leading to a worse clinical outcome. These effects of Lp(a) could be ascribed to the multiple detrimental effects that Lp(a) exerts on the vascular wall. Recent evidence of a longitudinal relationship of Lp(a) levels with the cardiovascular outcome in a large multiethnic cohort of hypertensive patients reinforces the need for more extensive clinical research in this field. This is further encouraged by the very promising results of the studies that have employed novel RNA-targeted treatments for Lp(a) reduction. If their benefits will be confirmed by the ongoing outcome trials, these new treatments will shift the gear for effective intervention on the residual cardiovascular risk of patients with high BP.

## Figures and Tables

**Table 1:** Mechanisms potentially involved in the proatherogenic effects of Lp(a).

General Vascular	Endothelial Cells	Vascular Smooth Muscle Cells
Passive diffusion to subendothelial space	Enhanced expression of adhesion molecules	Promotion of cell migration to subintima
Binding to proteoglycans by the lysin-binding pocket of kringle IV type-10	Accelerated senescence with disruption of cells	Integrin αVβ3-mediated activation of tyrosine kinase and RhoA
Interaction with oxidized phospholipids	Enhanced contraction and loss of contact between cells	Cell spreading with impaired cell remodeling and fiber formation
Increased generation of reactive oxygen species	Impaired adhesion and migration of endothelial progenitor cells	Vascular stiffening
	Impaired angiogenesis signaling pathways	
	Increased apoptosis	

## Data Availability

Not applicable.

## References

[1] Berg K. (1963). A new serum type system in man-the Lp(a) system. Acta Pathol. Microbiol. Scand..

[2] Dahlen G.H., Ericson C., Furberg C. (1972). Myocardial infarction and an extra pre-beta lipoprotein fraction. Acta Medica Scand..

[3] Walton K.W., Hithens J., Magnani H.N., Khan M. (1974). A study of methods of identification and estimation of Lp(a) lipoprotein and of its significance in health, hyperlipidaemia and atherosclerosis. Atherosclerosis.

[4] Kostner G.M., Avogaro P., Cazzolato G., Marth E., Bittolo-Bon G., Qunici G.B. (1981). Lipoprotein Lp(a) and the risk for myocardial infarction. Atherosclerosis.

[5] Armstrong V.W., Cremer P., Eberle E., Manke A., Shulze F., Wieland H., Kreuzer H., Seidel D. (1986). The association between serum Lp(a) concentrations and angiographically assessed coronary atherosclerosis. Atherosclerosis.

[6] Rhoads G.G., Dahlen G., Berg K., Morton N.E., Dannenberg A.L. (1986). Lp(a) lipoprotein as a risk factor for myocardial infarction. JAMA.

[7] Durrington P.N., Ishola M., Hunt L., Arrol S., Bhatnagar D. (1988). Apolipoproteins (a), AI and B and parental history in men with early onset ischaemic heart disease. Lancet.

[8] Hoefler G., Harnoncourt F., Paschke E., Mirtl W., Pfeiffer K.H., Kostner G.M. (1988). Lipoprotein Lp(a): A risk factor for myocardial infarction. Arteriosclerosis.

[9] Mbewu A.D., Durrington P.N. (1990). Lipoprotein (a): Structure, properties and possible involvement in thrombogenesis and atherogenesis. Atherosclerosis.

[10] Genest J., McNamara J.R., Ordovas J.M., Jenner J.L., Silberman S.R., Anderson K.M., Wilson P.W., Salem D.N., Schaefer E.J. (1992). Lipoprotein cholesterol, apolipoprotein A-I and B and lipoprotein (a) abnormalities in men with premature coronary artery disease. J. Am. Coll. Cardiol..

[11] Abe A., Noma A., Lee Y.J., Yamaguchi H. (1992). Studies on apolipoprotein (a) phenotypes Part 2 Phenotype frequen-cies and Lp(a) concentrations in different phenotypes in patients with anglographically defined coronary artery dis-ease. Atherosclerosis.

[12] Labeur C., De Bacquer D., De Backer G., Vincke J., Muyldermans L., Vandekerckhove Y., Van der Stichele E., Rosseneu M. (1992). Plasma lipoprotein (a) values and severity of eoronary artery disease in a large population of patients undergoing coronary angiography. Clin. Chem..

[13] Rosengren A., Wilhelmsen L., Eriksson E., Wedel H. (1990). Lipoprotein (a) and coronary heart disease: A prospective case-control study in a general population sample of middle-aged men. BMJ.

[14] Jauhiainen M., Koskinen P., Ehnholm C., Frick H.M., Manttari M., Manninen V., Huttunen J.K. (1991). Lipoprotein(a) and coronary heart disease risk: A nested case-control study of the Helsinki Heart Study participants. Atherosclerosis.

[15] Sigurdsson G., Baldursdottir A., Sigvaldason H., Agnarsson U., Thorgeirsson G., Sigfusson N. (1992). Predictive value of apolipoproteins in a prospective survey of coronary artery disease in men. Am. J. Cardiol..

[16] Cressman M.D., Heyka R.J., Paganini E.P., O’Neil J., Skibinski C.I., Hoff H.F. (1992). Lipoprotein(a) is an independent risk factor for cardiovascular disease in hemodialysis patients. Circulation.

[17] Alfthan G., Pekkanen J., Jauhiainen M., Pitkdniemi J., Karvonen M., Tuomilehto J., Salonen J.T., Ehnholm C. (1994). Relation of serum homocysteine and lipoprotein(a) concentrations to atherosclerotic disease in a prospective Finnish population based study. Atherosclerosis.

[18] Wald N.J., Law M., Watt H.C., Wu T., Bailey A., Johnson A.M., Craig W.Y., Ledue T.B., Haddow J.E. (1994). Apolipoproteins and ischaemic heart disease: Implications for screening. Lancet.

[19] Bostom A.G., Gagnon D.R., Cupples L.A., Wilson P.W., Jenner J.L., Ordovas J.M., Schaefer E.J., Castelli W.P. (1994). A prospective investigation of elevated lipoprotein (a) detected by electrophoresis and cardiovascular disease in women. The Framingham Heart Study. Circulation.

[20] Cremer P., Nagel D., Labrot B., Mann H., Muche R., Elster H., Seidel D. (1994). Lipoprotein Lp(a) as a predictor of myocardial infarction in comparison to fibrinogen, LDL cholesterol and other risk factors: Results from the prospective Gottingen Risk Incidence and Preva-lence Study (GRIPS). Eur. J. Clin. Investig..

[21] Schaefer E.J., Lamon-Fava S., Jenner J.L., McNamara J.R., Ordovas J.M., Davis C.E., Abolafia J.M., Lippel K., Levy R.I. (1994). Lipoprotein (a) levels and risk of coronary heart disease in men. JAMA.

[22] Haffner S.M., Moss S.E., Klein B.E.K., Klein R. (1992). Lack of association between lipoprotein (a) concentrations and coronary heart disease mortality in diabetes: The Wisconsin Epidemiologic Study of Diabetic Retinopathy. Metabolism.

[23] Winocour P.H., Durrington P.N., Bhatnagar D., Mbewu A.D., Ishola M., Mackness M., Arrol S. (1992). A cross-sectional evaluation of cardiovascular risk factors in coronary heart diseae associated with type 1 (insulin-dependent) diabetes mellitus. Diabetes Res. Clin. Pract..

[24] Ridker P.M., Hennekens C.H., Stampfer M.J. (1993). A prospective study of lipoprotein (a) and the risk of myocardial infarction. JAMA.

[25] Craig W.Y., Neveux L.M., Palomaki G.E., Cleveland M.M., Haddow J.E. (1998). Lipoprotein(a) as a risk factor for ischemic heart disease: Metaanalysis of prospective studies. Clin. Chem..

[26] Danesh J., Collins R., Peto R., Lipoprotein(a) and coronary heart disease (2000). Meta-analysis of prospective studies. Circulation.

[27] Emerging Risk Factors Collaboration (2009). Erqou, S.; Kaptoge, S.; Perry, P.L.; Di Angelantonio, E.; Thompson, A.; White, I.R.; Marcovina, S.M.; Collins, R.; Thompson, S.G.; et al. Lipoprotein(a) concentration and the risk of coronary heart disease, stroke, and nonvascular mortality. JAMA.

[28] Nave A.H., Lange K.S., Leonards C.O., Siegerink B., Doehner W., Landmesser U., Steinhagen-Thiessen E., Endres M., Ebinger M. (2015). Lipoprotein (a) as a risk factor for ischemic stroke: A meta-analysis. Atherosclerosis.

[29] Erqou S., Thompson A., Di Angelantonio E., Saleheen D., Kaptoge S., Marcovina S., Danesh J. (2010). Apolipoprotein(a) isoforms and the risk of vascular disease: Systematic review of 40 studies involving 58,000 participants. J. Am. Coll. Cardiol..

[30] Sultan S.M., Schupf N., Dowling M.M., Deveber G.A., Kirton Elkind M.S. (2014). Review of lipid and lipoprotein(a) abnormalities in childhood arterial ischemic stroke. Int. J. Stroke.

[31] Pare G., Caku A., McQueen M., Anand D.S., Enas E., Clarke R., Boffa M.B., Koschinsky M., Wang X., Yusuf S. (2019). Lipoprotein(a) levels and the risk of myocardial infarction among 7 ethnic groups. Circulation.

[32] Kamstrup P.R., Benn M., Tybjaerg-Hansen A., Nordestgaard B.G. (2008). Extreme lipoprotein(a) levels and risk of myocardial infarction in the general population: The Copenhagen City Heart Study. Circulation.

[33] Kamstrup P.R., Tybjaerg-Hansen A., Steffensen R., Nordestgaard B.G. (2009). Genetically elevated lipoprotein(a) and increased risk of myocardial infarction. JAMA.

[34] Kamstrup P.R., Tybjaerg-Hansen A., Nordestgaard B.G. (2014). Elevated lipoprotein(a) and risk of aortic valve stenosis in the general population. J. Am. Coll. Cardiol..

[35] Kamstrup P.R., Tybjaerg-Hansen A., Nordestgaard B.G. (2012). Genetic evidence that lipoprotein(a) associates with atherosclerotic stenosis rather than venous thrombosis. Arterioscler. Thromb. Vasc. Biol..

[36] Kamstrup P.R., Nordestgaard B.G. (2016). Elevated lipoprotein(a) levels, LPA risk genotypes, and increased risk of heart failure in the general population. JACC Heart Fail..

[37] Langsted A., Kamstrup P.R., Nordestgaard B.G. (2019). High lipoprotein(a) and high risk of mortality. Eur. Heart J..

[38] Langsted A., Nordestgaard B.G., Kamstrup P.R. (2019). High lipoprotein(a) and increased risk of ischemic stroke in a large contemporary general population study. J. Am. Coll. Cardiol..

[39] Steffen B., Duprez D., Bertoni A., Guan W., Tsai M. (2018). Lp(a) [Lipoprotein(a)]-related risk of heart failure is evident in whites but not in other racial/ethnic groups. Arterioscler. Thromb. Vasc. Biol..

[40] Smith G.D., Ebrahim S. (2003). ‘Mendelian randomization’: Can genetic epidemiology contribute to understanding environmental determinants of disease?. Int. J. Epidemiol..

[41] Smith G.D., Ebrahim S., Lewis S., Hansell A.L., Palmer L.J., Burton P.R. (2005). Genetic epidemiology and public health: Hope, hype, and future prospects. Lancet.

[42] Benn M., Nordestgaard B.G. (2018). From genome-wide association studies to mendelian randomization: Novel opportunities for understanding cardiovascular disease causality, pathogenesis, prevention, and treatment. Cardiovasc. Res..

[43] Clarke R., Peden J.F., Hopewell J.C., Kyriakou T., Goel A., Heath S.C., Parish S., Barlera S., Franzosi M.G., Rust S. (2009). Genetic variants associated with Lp(a) lipoprotein level and coronary disease. N. Engl. J. Med..

[44] Tregouet D.A., Konig I.R., Erdmann J., Munteanu A., Braund P.S., Hall A.S., Grosshennig A., Linsel-Nitschke P., Perret C., DeSuremain M. (2009). Genome-wide haplotype association study identifies the SLC22A3-LPAL2-LPA gene cluster as a risk locus for coronary artery disease. Nat. Genet..

[45] Schunkert H., Konig I.R., Kathiresan S., Reilly M.P., Assimes T.L., Holm H., Preuss M., Stewart A.F.R., Barbalic M., Gieger C. (2011). Large-scale association analysis identifies 13 new susceptibility loci for coronary artery disease. Nat. Genet..

[46] Thanassoulis G., Campbell C.Y., Owens D.S., Smith J.G., Smith A.V., Peloso G.M., Kerr K.F., Pechlivanis S., Budoff M.J., Harris T.B. (2013). Genetic associations with valvular calcification and aortic stenosis. N. Engl. J. Med..

[47] Helgadottir A., Thorleifsson G., Gretarsdottir S., Stefansson O.A., Tragante V., Thorolfsdottir R.B., Jonsdottir I., Bjornsson T., Steinthorsdottir V., Verweij N. (2018). Genome-wide analysis yields new loci associating with aortic valve stenosis. Nat. Commun..

[48] Willeit P., Kiechl S., Kronenberg F., Witztum J.L., Santer P., Mayr M., Xu Q., Mayr A., Willeit J., Tsimikas S. (2014). Discrimination and net reclassification of cardiovascular risk with lipoprotein(a): Prospective 15-year outcomes in the Bruneck study. J. Am. Coll. Cardiol..

[49] Albers J.J., Slee A., O’Brien K.D., Robinson J.G., Kashyap M.L., Kwiterovich P.O., Xu P., Marcovina S.M. (2013). Relationship of apolipoproteins A-1 and B, and lipoprotein(a) to cardiovascular outcomes: The AIM-HIGH trial (Atherothrombosis intervention in metabolic syndrome with low HDL/high triglyceride and impact on global health outcomes). J. Am. Coll. Cardiol..

[50] Khera A.V., Everett B.M., Caulfield M.P., Hantash F.M., Wohlgemuth J., Ridker P.M., Mora S. (2014). Lipoprotein(a) concentrations, rosuvastatin therapy, and residual vascular risk: An analysis from the JUPITER trial (justification for the use of statins in prevention: An intervention trial evaluating rosuvastatin). Circulation.

[51] Nestel P.J., Barnes E.H., Tonkin A.M., Simes J., Fournier M., White H.D., Colquhoun D.M., Blankenberg S., Sullivan D.R. (2013). Plasma lipoprotein(a) concentration predicts future coronary and cardiovascular events in patients with stable coronary heart disease. Arterioscler. Thromb. Vasc. Biol..

[52] Grundy S.M., Stone N.J., Bailey A.L., Beam C., Birtcher K.K., Blumenthal R.S., Braun L.T., De Ferranti S., Faiella-Tommasino J., Forman D.E. (2019). 2018 AHA/ACC/AACVPR/AAPA/ABC/ACPM/ADA/AGS/AphA/ASPC/NLA/PCNA Guideline on the Management of Blood Cholesterol: A Report of the American College of Cardiology/American Heart Association Task Force on Clinical Practice Guidelines. J. Am. Coll. Cardiol..

[53] Stefanutti C., Julius U., Watts G.F., Harada-Shiba M., Cossu M., Schettler V.J., De Silvestro G., Soran H., Van Lennep J.R., Pisciotta L. (2017). Toward an international consensus-Integrating lipoprotein apheresis and new lipid-lowering drugs. J. Clin. Lipidol..

[54] Anderson T.J., Gregoire J., Pearson G.J., Barry A.R., Couture P., Dawes M., Francis G.A., Genest J., Grover S., Gupta M. (2016). 2016 Canadian Cardiovascular Society Guidelines for the management of dyslipidemia for the prevention of cardiovascular disease in the adult. Can. J. Cardiol..

[55] Wilson D.P., Jacobson T.A., Jones P.H., Koschinsky M.L., McNeal C.J., Nordestgaard B.G., Orringer C.E. (2019). Use of Lipoprotein(a) in clinical practice: A biomarker whose time has come. A scientific statement from the National Lipid Association. J. Clin. Lipidol..

[56] Cegla J., Neely R.D.G., France M., Ferns G., Byrne C.D., Halcox J., Datta D., Capps N., Shoulders C., Qureshi N. (2019). HEART UK consensus statement on Lipoprotein(a): A call to action. Atherosclerosis.

[57] Tsimikas S. (2017). A Test in Context: Lipoprotein(a): Diagnosis, Prognosis, Controversies, and Emerging Therapies. J. Am. Coll. Cardiol..

[58] Tsimikas S., Viney N.J., Hughes S.G., Singleton W., Graham M.J., Baker B.F., Burkey J.L., Yang Q., Marcovina S.M., Geary R.S. (2015). Antisense therapy targeting apolipoprotein(a): A randomised, double-blind, placebo-controlled phase 1 study. Lancet.

[59] Viney N.J., van Capelleveen J.C., Geary R.S., Xia S., Tami J.A., Yu R.Z., Marcovina S.M., Hughes S.G., Graham M.J., Crooke R.M. (2016). Antisense oligonucleotides targeting apolipoprotein(a) in people with raised lipoprotein(a): Two randomised, double-blind, placebocontrolled, dose-ranging trials. Lancet.

[60] Tsimikas S., Fazio S., Ferdinand K.C., Ginsberg H.N., Koschinsky M.L., Marcovina S.M., Moriarty P.M., Rader D.J., Remaley A.T., Reyes-Soffer G. (2018). NHLBI Working Group Rec-ommendations to Reduce Lipoprotein(a)-Mediated Risk of Cardiovascular Disease and Aortic Stenosis. J. Am. Coll. Cardiol..

[61] Williams B., Mancia G., Spiering W., Agabiti Rosei E., Azizi M., Burnier M., Clement D.L., Coca A., de Simone G., Dominiczak A. (2018). 2018 ESC/ESH Guidelines for the management of arterial hypertension. Eur. Heart J..

[62] Whelton P.K., Carey R.M., Aronow W.S., Casey D.E.J., Collins K.J., Dennison Himmelfarb C., DePalma S.M., Gidding S., Jamerson K.A., Jones D.W. (2018). 2017 ACC/AHA/AAPA/ABC/ACPM/AGS/APhA/ASH/ASPC/NMA/PCNA Guideline for the Prevention, Detection, Evaluation, and Management of High Blood Pressure in Adults: A Report of the American College of Cardiology/American Heart Association Task Force on Clinical Practice Guidelines. Hypertension.

[63] World Health Organization [WHO] (2016). World Health Organization Obesity and Overweight Fact Sheet 2016.

[64] Lim S.S., Vos T., Flaxman A.D., Danaei G., Shibuya K., Adair-Rohani H., Amann M., Anderson H.R., Andrews K.G., Aryee M. (2012). A comparative risk assessment of burden of disease and injury attributable to 67 risk factors and risk factor clusters in 21 regions, 1990–2010: A systematic analysis for the Global Burden of Disease Study 2010. Lancet.

[65] Sechi L.A., Kronenberg F., De Carli S., Falleti E., Zingaro L., Catena C., Utermann G., Bartoli E. (1997). Association of lipoprotein(a) levels and apolipoprotein(a) polymorphism with target-organ damage in arterial hypertension. JAMA.

[66] Brosolo G., Da Porto A., Bulfone L., Vacca A., Bertin N., Colussi G., Cavarape A., Sechi L.A., Catena C. (2021). Plasma lipoprotein(a) levels as determinants of arterial stiffening in hypertension. Biomedicines.

[67] Gordon Hguyatt G.H., Oxman A.D., Vist G.E., Kunz R., Falck-Ytter Y., Alonso-Coello P., Schünemann H.J. (2008). GRADE: An emerging consensus on rating quality of evidence and strength of recommendations. BMJ.

[68] Boffa M.B., Koschinsky M.L. (2016). Lipoprotein (a): Truly a direct prothrombotic factor in cardiovascular disease?. J. Lipid Res..

[69] Utermann G., Weber W. (1983). Protein composition of Lp(a) lipoprotein from human plasma. FEBS (Fed. Eur. Biochem. Soc.).

[70] Albers J.J., Kenedy H., Marcovìna S.M. (1996). Evidence that Lp(a) contains one molecole of apo(a) and one molecule of apo B: Evaluation of amino acid analysis data. J. Lipid Res..

[71] Sommer A., Prenner E., Gorges R., Stutz H., Grillhofer H., Kostner G.M., Paltauf F., Hermetter A. (1992). Organization of phosphatidylcholine and sphingomyelin in the surface monolayer of low density lipoprotein and lipoprotein(a) as determined by time-resolved fluorometry. J. Biol. Chem..

[72] Frank S.L., Klisak I., Sparkes R.S., Mohandas T., Tomlinson J.E., McLean J.W., Lawn R.M., Lusis A.J. (1988). The apolipoprotein(a) gene resides on human chromosome 6q26-27, in close proximity to the homologous gene for plasminogen. Hum. Genet..

[73] McLean J.W., Tomlinson J.E., Kuang W.J., Eaton D.L., Chen E.T., Fless G.M., Scanu A.M., Lawn R.M. (1987). cDNA sequence of human apolipoprotein(a) is homologous to plasminogen. Nature.

[74] Marcovina M., Albers J.J., Gabel B., Koschinsky M.L., Gaur V.P. (1995). Effect of the number of apolipoprotein (a) kringle 4 domains on immunochemical measurements of lipoprotein(a). Clin. Chem..

[75] Utermann G. (1989). The mysteries of lipoprotein(a). Science.

[76] Forsgren M., Raden B., Israelsson M., Larsson K., Heden L.O. (1987). Molecular cloning and characterization of a full-length cDNA clone for human plasminogen. FEBS Lett..

[77] Koschinsky M.L., Boffa M.B., Marcovina S.M., Ballantyne C.M. (2015). Lipoprotein(a) in cardiovascular risk assessment. Clinical Lipidology: A Companion to Braunwald’s Heart Disease.

[78] Harpel P.C., Gordon B.R., Parker T.S. (1989). Plasmin catalyzes binding of lipoprotein (a) to immobilized fibrinogen and fibrin. Proc. Natl. Acad. Sci. USA.

[79] Hoover-Plow J., Huang M. (2013). Lipoprotein(a) metabolism: Potential sites for therapeutic targets. Metabolism.

[80] Cai A., Li L., Zhang Y., Mo Y., Mai W., Zhou Y. (2013). Lipoprotein(a): A promising marker for residual cardiovascular risk assessment. Dis. Markers.

[81] Leibundgut G., Scipione C., Yin H., Schneider M., Boffa M.B., Green S., Yang X., Dennis E., Witztum J.L., Koschinsky M.L. (2013). Determinants of binding of oxidized phospholipids on apolipoprotein (a) and lipoprotein (a). J. Lipid Res..

[82] Scipione C.A., Sayegh S.E., Romagnuolo R., Tsimikas S., Marcovina S.M., Boffa M.B., Koschinsky M.L. (2015). Mechanistic insights into Lp(a)-induced IL-8 expression: A role for oxidized phospholipid modification of apo(a). J. Lipid Res..

[83] Taleb A., Witztum J.L., Tsimikas S. (2011). Oxidized phospholipids on apoB-100-containing lipoproteins: A biomarker predicting cardiovascular disease and cardiovascular events. Biomarkers Med..

[84] Ernst A., Helmhold M., Brunner C., Pethö-Schramm A., Armstrong V.W., Müller H.J. (1999). Identification of two functionally distinct lysine-binding sites in kringle 37 and in kringles 32–36 of human apolipoprotein(a). J. Biol. Chem..

[85] Gabel B.R., May L.F., Marcovina S.M., Koschinsky M.L. (1996). Lipoprotein(a) assembly. Quantitative assessment of the role of apo(a) kringle IV types 2–10 in particle formation. Arterioscler. Thromb. Vasc. Biol..

[86] Patthy L. (1985). Evolution of the proteases of blood coagulation and fibrinolysis by assembly from modules. Cell.

[87] von Zychlinski A., Kleffmann T., Williams M.J.A., McCormick S.P. (2011). Proteomics of Lipoprotein(a) identifies a protein complement associated with response to wounding. J. Proteom..

[88] von Zychlinski A., Williams M., McCormick S., Kleffmann T. (2014). Absolute quantification of apolipoproteins and associated proteins on human plasma lipoproteins. J. Proteom..

[89] Damluji A.A., El-Maouche D., Alsulaimi A., Martin P., Shamburek R.D., Goldberg R.B., Baum S.J., de Marchen E.J. (2016). Accelerated atherosclerosis and elevated lipoprotein (a) after liver transplantation. J. Clin. Lipidol..

[90] Barbir M., Khaghani A., Kehely A., Tan K.C., Mitchell A., Thompson G.R., Yacoub M. (1992). Normal levels of lipoproteins including lipoprotein(a) after liver- heart transplantation in a patient with homozygous familial hypercholesterolaemia. Q. J. Med..

[91] Lawn R.M., Schwartz K., Patthy L. (1997). Convergent evolution of apolipoprotein(a) in primates and hedgehog. Proc. Natl. Acad. Sci. USA.

[92] Haibach C., Kraft H.G., Köchl S., Abe A., Utermann G. (1998). The number of kringle IV repeats 3–10 is invariable in the human apo(a) gene. Gene.

[93] Erdel M., Hubalek M., Lingenhel A., Kofler K., Duba H.C., Utermann G. (1999). Counting the repetitive kringle-IV repeats in the gene encoding human apolipoprotein(a) by fibre-FISH [letter]. Nat. Genet..

[94] Berg K. (1968). The Lp system. Ser. Haematol..

[95] Kronenberg F. (2016). Human genetics and the causal role of lipoprotein(a) for various diseases. Cardiovasc. Drugs Ther..

[96] Laschkolnig A., Kollerits B., Lamina C., Meisinger C., Rantner B., Stadler M., Peters A., Koenig W., Stöckl A., Dähnhardt D. (2014). Lipoprotein (a) concentrations, apolipoprotein (a) phenotypes, and peripheral arterial disease in three independent cohorts. Cardiovasc. Res..

[97] Kronenberg F. (2014). Genetic determination of lipoprotein(a) and its association with cardiovascular disease: Convenient does not always mean better. J. Intern. Med..

[98] Guadagno P.A., Summers Bellin E.G., Harris W.S., Dayspring T.D., M Hoefner D.M., Thiselton D.L., Stanovick B., Warnick G.R., McConnell J.P. (2015). Validation of a lipoprotein(a) particle concentration assay by quantitative lipoprotein immunofixation electrophoresis. Clin. Chim. Acta.

[99] Brunner C., Lobentanz E.M., Petho-Schramm A., Ernst A., Kang C., Dieplinger H., Muller H.J., Utermann G. (1996). Thè number of identical kringle IV repeats in apolipoprotein(a) affects rts processing and secretion by HepG2 cells. J. Biol. Chem..

[100] Lobentanz E.M., Krasznai K., Gruber A., Brunner C., Muller H.J., Sattler J., Kraft H.G., Utermann G., Dieplinger H. (1998). Intracellular metabolism of human apolipoprotein (a) in stably transfected Hep G2 cells. Biochemistry.

[101] White A.L., Hixson J.E., Rainwater D.L., Lanford R.E. (1994). Molecular basis for “null” lipoprotein(a) phenotypes and the influence of apolipoprotein(a) size on plasma lipoprotein(a) level in the baboon. J. Biol. Chem..

[102] Frazer K.A., Narla G., Zhang J.L., Rubin E.M. (1995). The apolipoprotein(a) gene is regulated by sex hormones and acute-phase inducers in YAC transgenic mice. Nat. Genet..

[103] Wade D.P., Puckey L.H., Knight B.L., Acquati F., Mihalich A., Taramelli R. (1997). Characterization of multiple enhancer regions upstream of the apolipoprotein(a) gene. J. Biol. Chem..

[104] Boffelli D., Zajchowski D.A., Yang Z., Lawn R.M. (1999). Estrogen modulation of apolipoprotein(a) expression: Identification of a regulatory element. J. Biol. Chem..

[105] Huby T., Afzal V., Doucet C., Lawn R.M., Gong E.L., Chapman M.J., Thillet J., Rubin E.M. (2003). Regulation of the expression of the apolipoprotein(a) gene: Evidence for a regulatory role of the 5′ distal apolipoprotein(a) transcription control region enhancer in yeast artificial chromosome transgenic mice. Arterioscler. Thromb. Vasc. Biol..

[106] Negi S., Singh S.K., Pati N., Handa V., Chauhan R., Pati U. (2004). A proximal tissue-specific module and a distal negative module control apolipoprotein(a) gene transcription. Biochem. J..

[107] Wade D.P., Lindahl G.E., Lawn R.M. (1994). Apolipoprotein(a) gene transcription is regulated by liver-enriched trans-acting factor hepatocyte nuclear factor alpha. J. Biol. Chem..

[108] Handa V., Mahboob-ul-hussain, Pati N., Pati U. (2002). Multiple liver-specific factors bind to a 64-bp element and activate apo(a) gene. Biochem. Biophys. Res. Commun..

[109] Kagawa A., Azuma H., Akaike M., Kanagawa Y., Matsumoto T. (1999). Aspirin reduces apolipoprotein(a) (apo(a)) production in human hepatocytes by suppression of apo(a) gene transcription. J. Biol. Chem..

[110] Mooser V., Mancini F.P., Bopp S., Pethö-Schramm A., Guerra R., Boerwinkle E., Müller H.J., Hobbs H.H. (1995). Sequence polymorphisms in the apo(a) gene associated with specific levels of Lp(a) in plasma. Hum. Mol. Genet..

[111] Trommsdorff M., Köchl S., Lingenhel A., Kronenberg F., Delport R., Vermaak H., Lemming L., Klausen I.C., Faergeman O., Utermann G. (1995). A pentanucleotide repeat polymorphism in the 5′ control region of the apolipoprotein(a) gene is associated with lipoprotein(a) plasma concentrations in Caucasians. J. Clin. Investig..

[112] Kostner K.M., Kostner G.M., Wierzbicki A.S. (2018). Is Lp(a) ready for prime time use in the clinic? A pros-and-cons debate. Atherosclerosis.

[113] Hung M.Y., Tsimikas S. (2014). What is the ultimate test that lowering lipoprotein(a) is beneficial for cardiovascular disease and aortic stenosis?. Curr. Opin. Lipidol..

[114] Boerwinkle E., Leffert C.C., Lin J., Lackner C., Chiesa G., Hobbs H.H. (1992). Apolipoprotein(a) gene accounts for greater than 90% of the variation in plasma lipoprotein(a) concentrations. J. Clin. Investig..

[115] Schmidt K., Noureen A., Kronenberg F., Utermann G. (2016). Structure, function, and genetics of lipoprotein (a). J. Lipid Res..

[116] Nordestgaard B.G., Langsted A. (2016). Lipoprotein (a) as a cause of cardiovascular disease: Insights from epidemiology, genetics, and biology. J. Lipid Res..

[117] Hopewell J.C., Clarke R., Parish S., Armitage J., Lathrop M., Hager J., Collins R., Heart Protection Study Collaborative Group (2011). Lipoprotein(a) genetic variants associated with coronary and peripheral vascular disease but not with stroke risk in the Heart Protection Study. Circ. Cardiovasc. Genet..

[118] Luke M.M., Kane J.P., Liu D.M., Rowland C.M., Shiffman D., Cassano J., Catanese J.J., Pullinger C.R., Leong D.U., Arellano A.R. (2007). A polymorphism in the protease-like domain of apolipoprotein(a) is associated with severe coronary artery disease. Arterioscler. Thromb. Vasc. Biol..

[119] Arai K., Luke M.M., Koschinsky M.L., Miller E.R., Pullinger C.R., Witztum J.L., Kane J.P., Tsimikas S. (2010). The I4399M variant of apolipoprotein(a) is associated with increased oxidized phospholipids on apolipoprotein B-100 particles. Atherosclerosis.

[120] Lanktree M.B., Anand S.S., Yusuf S., Hegele R.A. (2010). Comprehensive analysis of genomic variation in the LPA locus and its relationship to plasma lipoprotein(a) in South Asians, Chinese, and European Caucasians. Circ. Cardiovasc. Genet..

[121] Ronald J., Rajagopalan R., Cerrato F., Nord A.S., Hatsukami T., Kohler T., Marcovina S., Heagerty P., Jarvik G.P. (2011). Genetic variation in LPAL2, LPA, and PLG predicts plasma lipoprotein(a) level and carotid artery disease risk. Stroke.

[122] Deo R.C., Wilson J.G., Xing C., Lawson K., Kao W.H., Reich D., Tandon A., Akylbekova E., Patterson N., Mosley T.H. (2011). Single-nucleotide polymorphisms in LPA explain most of the ancestry-specific variation in Lp(a) levels in African Americans. PLoS ONE.

[123] Kostner K.M., Kostner G.M. (2017). Lipoprotein (a): A historical appraisal. J. Lipid Res..

[124] Tsimikas S., Moriarty P.M., Stroes E.S. (2021). Emerging RNA therapeutics to lower blood levels of Lp(a): JACC focus seminar 2/4. J. Am. Coll. Cardiol..

[125] Yeang C., Gordts P.L., Tsimikas S. (2017). Novel Lipoprotein(a) catabolism pathway via apolipoprotein(a) recycling: Adding the plasminogen receptor PlgR (KT) to the List. Circ. Res..

[126] Sharma M., Redpath G.M., Williams M.J.A., McCormick S.P.A. (2017). Recycling of apolipoprotein(a) after PlgRKT-mediated endocytosis of lipoprotein(a). Circ. Res..

[127] Nordestgaard B.G., Chapman M.J., Ray K., Boren J., Andreotti F., Watts G.F., Ginsberg H., Amarenco P., Catapano A., Descamps O.S. (2010). Lipoprotein(a) as a cardiovascular risk factor: Current status. Eur. Heart J..

[128] Tsimikas S., Stroes E.S.G. (2020). The dedicated “Lp(a) clinic”: A concept whose time has arrived?. Atherosclerosis.

[129] Ellis K.L., Boffa M.B., Sahebkar A., Koschinsky M.L., Watts G.F. (2017). The renaissance of lipoprotein(a): Brave new world for preventive cardiology?. Prog. Lipid Res..

[130] Sechi L.A., Zingaro L., De Carli S., Sechi G., Catena C., Falleti E., Dell’Anna E., Bartoli E. (1998). Increased serum lipoprotein(a) levels in patients with early renal failure. Ann. Intern. Med..

[131] Scipione C.A., Koschinsky M.L., Boffa M.B. (2018). Lipoprotein(a) in clinical practice: New perspectives from basic and translational science. Crit. Rev. Clin. Lab. Sci..

[132] Dati F., Tate J.R., Marcovina S.M., Steinmetz A., International Federation of Clinical Chemistry and Laboratory Medicine, IFCC Working Group for Lipoprotein(a) Assay Standardization (2004). First WHO/IFCC International Reference Reagent for Lipoprotein(a) for Immunoassay-Lp(a) SRM 2B. Clin. Chem. Lab. Med..

[133] Marcovina S.M., Albers J.J., Scanu A.M., Kennedy H., Giaculli F., Berg K., Couderc R., Dati F., Rifai N., Sakurabayashi I. (2000). Use of a reference material proposed by the International Federation of Clinical Chemistry and Laboratory Medicine to evaluate analytical methods for the determination of plasma lipoprotein(a). Clin. Chem..

[134] Marcovina S.M., Clouet-Foraison N., Koschinsky M.L., Lowenthal M.S., Orquillas A., Boffa M.B., Hoofnagle A.N., Vaisar T. (2021). Development of an LC-MS/MS proposed candidate reference method for the standardization of analytical methods to measure lipoprotein(a). Clin. Chem..

[135] Borghi C. (2002). Interactions between hypercholesterolemia and hypertension: Implications for therapy. Curr. Opin. Nephrol. Hypertens..

[136] Gebrie A., Gnanasekaran N., Menon M., Sisay M., Zegeye A. (2018). Evaluation of lipid profiles and hematological parameters in hypertensive patients: Laboratory-based cross-sectional study. SAGE Open Med..

[137] Gazzaruso C., Buscaglia P., Garzaniti A., Bonetti G., Savino S., Mariotti S., Jucci A., Finardi G., Geroldi D. (1996). Lipoprotein(a) plasma concentrations, apolipoprotein (a) polymorphism and family history of coronary heart disease in patients with essential hypertension. J. Cardiovasc. Risk.

[138] Constans J., Wendling G., Peuchant E., Camilleri G., Conri C. (1996). Lipoprotein(a) in 505 hospitalized patients with various pathological states: Correlations with cardiovascular diseases and therapies. Int. Angiol..

[139] Saku K., Liu K., Takeda Y., Jimi S., Arakawa K. (1995). Effects of lisinopril and bisoprolol on lipoprotein metabolism in patients with mild-to-moderate essential hypertension. Clin. Ther..

[140] Van Wersch J.W. (1994). The behaviour of lipoprotein(a) in patients with various diseases. Scand. J. Clin. Lab. Investig..

[141] Glueck C.J., Glueck H.I., Hamer T., Speirs J., Tracy T., Stroop D. (1994). Beta blockers, Lp(a), hypertension, and reduced basal fibrinolytic activity. Am. J. Med. Sci..

[142] Zhuang Y.Y., Wang J.J., Xu P. (1993). Increased lipoprotein (a) as an independent risk factor for cardiovascular and cerebro-vascular diseases. Chin. Med. J..

[143] Catalano M., Perilli E., Carzaniga G., Colombo F., Carotta M., Andreoni S. (1998). Lp(a) in hypertensive patients. J. Hum. Hypertens..

[144] Lip G.Y., Blann A.D., Jones A.F., Lip P.L., Beevers D.G. (1997). Relation of endothelium, thrombogenesis, and hemorheology in systemic hypertension to ethnicity and left ventricular hypertrophy. Am. J. Cardiol..

[145] Ward N.C., Nolde J.M., Chan J., Carnagarin R., Watts G.F., Schlaich M.P. (2021). Lipoprotein (a) and hypertension. Curr. Hypertens. Rep..

[146] Lima L.M., Carvalho M.d.G., Loures-Vale A.A., Fernandes A.P., Mota A.P., Neto C.P., Garcia J.C.F., Saad J.A., Souza M.D.O. (2006). Increased serum levels of lipoprotein (a) correlated with the severity of coronary artery disease in patients submitted to angiography. Arq. Bras. Cardiol..

[147] Mahto S.K., Sheoran A., Gadpayle A.K., Gupta K., Gupta P.K., Chitkara A., Agarwal N. (2022). Evaluation of lipoprotein (a) [Lp(a)] and lipid abnormalities in patients with newly detected hypertension and its association with severity of hypertension. J. Fam. Med. Prim. Care.

[148] Bhavani B.A., Padma T., Sastry B., Reddy N.K. (2003). Plasma Lipoprotein (a) levels in patients with untreated essential hyper-tension. Indian J. Hum. Genet..

[149] Coffman T.M. (2011). Under pressure: The search for the essential mechanisms of hypertension. Nat. Med..

[150] Alexander Y., Osto E., Schmidt-Trucksäss A., Shechter M., Trifunovic D., Duncker D.J., Aboyans V., Bäck M., Badimon L., Cosentino F. (2021). Endothelial function in cardiovascular medicine: A consensus paper of the European Society of Cardiology Working Groups on Atherosclerosis and Vascular Biology, Aorta and Peripheral Vascular Diseases, Coronary Pathophysiology and Microcirculation, and Thrombosis. Cardiovasc. Res..

[151] Brandes R.P. (2014). Endothelial dysfunction and hypertension. Hypertension.

[152] Konukoglu D., Uzun H. (2017). Endothelial dysfunction and hypertension. Adv. Exp. Med. Biol..

[153] Rajendran P., Rengarajan T., Thangavel J., Nishigaki Y., Sakthisekaran D., Sethi G., Nishigaki I. (2013). The vascular endothelium and human diseases. Int. J. Biol. Sci..

[154] Tsimikas S. (2019). Potential causality and emerging medical therapies for lipoprotein(a) and its associated oxidized phos-pholipids in calcific aortic valve stenosis. Circ. Res..

[155] Nielsen L.B., Grønholdt M.L., Schroeder T.V., Stender S., Nordestgaard B.G. (1997). In vivo transfer of lipoprotein(a) into human atherosclerotic carotid arterial intima. Arterioscler. Thromb. Vasc. Biol..

[156] Hoover-Plow J.L., Miles L.A., Fless G.M., Scanu A.M., Plow E.F. (1993). Comparison of the lysine binding functions of lipopro-tein(a) and plasminogen. Biochemistry.

[157] Bergmark C., Dewan A., Orsoni A., Merki E., Miller E.R., Shin M.J., Binder C.J., Hörkkö S., Krauss R.M., Chapman M.J. (2008). A novel function of lipoprotein [a] as a preferential carrier of oxidized phospholipids in human plasma. J. Lipid Res..

[158] Edelstein C., Pfaffinger D., Hinman J., Miller E., Lipkind G., Tsimikas S., Bergmark C., Getz G.S., Witztum J.L., Scanu A.M. (2003). Lysinephosphatidylcholine adducts in kringle V impart unique immunological and potential pro-inflammatory properties to human apolipoprotein(a). J. Biol. Chem..

[159] Allen S., Khan S., Tam S., Koschinsky M., Taylor P., Yacoub M. (1998). Expression of adhesion molecules by lp(a): A potential novel mechanism for its atherogenicity. FASEB J..

[160] Takami S., Yamashita S., Kihara S., Ishigami M., Takemura K., Kume N., Kita T., Matsuzawa Y. (1998). Lipoprotein(a) enhances the expression of intercellular adhesion molecule-1 in cultured human umbilical vein endothelial cells. Circulation.

[161] Iwabayashi M., Taniyama Y., Sanada F., Azuma J., Iekushi K., Okayama K., Chatterjee A., Rakugi H., Morishita R. (2012). Inhibition of Lp(a)-induced functional impairment of endothelial cells and endothelial progenitor cells by hepato-cyte growth factor. Biochem. Biophys. Res. Commun..

[162] Wei D.H., Zhang X.L., Wang R., Zeng J.F., Zhang K., Yang J., Li S., Lin X.L., Jiang Z.S., Wang G.X. (2013). Oxi-dized lipoprotein(a) increases endothelial cell monolayer permeability via ROS generation. Lipids.

[163] Cho T., Jung Y., Koschinsky M.L. (2008). Apolipoprotein(a), through its strong lysine-binding site in KIV(10’), mediates increased endothelial cell contraction and permeability via a Rho/Rho kinase/MYPT1-dependent pathway. J. Biol. Chem..

[164] Pellegrino M., Furmaniak-Kazmierczak E., LeBlanc J.C., Cho T., Cao K., Marcovina S.M., Boffa M.B., Côté G.P., Koschinsky M.L. (2004). The apolipoprotein(a) component of lipoprotein(a) stimulates actin stress fiber formation and loss of cell-cell contact in cultured endothelial cells. J. Biol. Chem..

[165] Wang R., Zhang K., Li S., Tong Z., Li G., Zhao Z., Zhao Y., Liu F., Lin X., Wang Z. (2013). Apolipoprotein (a) Impairs Endothelial Progenitor Cell-Mediated Angiogenesis. DNA Cell Biol..

[166] Kalaivani V., Jaleel A. (2020). Apolipoprotein(a), an enigmatic anti-angiogenic glycoprotein in human plasma: A curse or cure?. Pharmacol. Res..

[167] Moeslinger T., Friedl R., Volf I., Brunner M., Koller E., Spieckermann P.G. (2000). Inhibition of inducible nitric oxide synthesis by oxidized lipoprotein(a) in a murine macrophage cell line. FEBS Lett..

[168] Rubanyi G.M., Freay A.D., Lawn R.M. (2000). Endothelium-dependent vasorelaxation in the aorta of transgenic mice expressing human apolipoprotein(a) or lipoprotein(a). Endothelium.

[169] O’Neil C.H., Boffa M.B., Hancock M.A., Pickering J.G., Koschinsky M.L. (2004). Stimulation of vascular smooth muscle cell proliferation and migration by apolipoprotein(a) is dependent on inhibition of transforming growth factor-beta activation and on the presence of kringle IV type 9. J. Biol. Chem..

[170] Riches K., Franklin L., Maqbool A., Peckham M., Adams M., Bond J., Warburton P., Feric N.T., Koschinsky M.L., O’Regan D.J. (2013). Apolipoprotein(a) acts as a chemorepellent to human vascular smooth muscle cells via integrin αVβ3 and RhoA/ROCK-mediated mechanisms. Int. J. Biochem. Cell Biol..

[171] Sorensen K.E., Celermajer D.S., Georgakopoulos D., Hatcher G., Betteridge D.J., Deanfield J.E. (1994). Impairment of endothelium-dependent dilation is an early event in children with familial hypercholesterolemia and is related to the lipoprotein(a) level. J. Clin. Investig..

[172] Celermajer D.S. (1993). Non-Invasive Studies of Arterial Physiology in Children and Adults at Risk of Atherosclerosis. Ph.D. Thesis.

[173] Schlaich M.P., John S., Langenfeld M.R., Lackner K.J., Schmitz G., Schmieder R.E. (1998). Does lipoprotein(a) impair endothelial function?. J. Am. Coll. Cardiol..

[174] Tsurumi Y., Nagashima H., Ichikawa K.I., Sumiyoshi T., Hosoda S. (1995). Influence of plasma lipoprotein(a) levels on coronary vasomotor response to acetylcholine. J. Am. Coll. Cardiol..

[175] Schachinger V., Halle M., Minners J., Berg A., Zeiher A.M. (1997). Lipoprotein(a) selectively impairs receptor-mediated endothelial vasodilator function of the human coronary circulation. J. Am. Coll. Cardiol..

[176] Raitakari O.T., Pitkänen O.P., Lehtimäki T., Lahdenperä S., Iida H., Ylä-Herttuala S., Luoma J., Mattila K., Nikkari T., Taskinen M.R. (1997). In vivo low density lipoprotein oxidation relates to coronary reactivity in young men. J. Am. Coll. Cardiol..

[177] Raitakari O.T., Adams M.R., Celermajer D.S. (1999). Effect of Lp(a) on the early functional and structural changes of atherosclerosis. Arterioscler. Thromb. Vasc. Biol..

[178] Wu H.D., Berglund L., Dimayuga C., Jones J., Sciacca R.R., Di Tullio M.R., Homma S. (2004). High lipoprotein(a) levels and small apolipoprotein(a) sizes are associated with endothelial dysfunction in a multiethnic cohort. J. Am. Coll. Cardiol..

[179] Mancia G., Volpe R., Boros S., Ilardi M., Giannattasio C. (2004). Cardiovascular risk profile and blood pressure control in Italian hypertensive patients under specialist care. J. Hypertens..

[180] Iannuzzo G., Tripaldella M., Mallardo V., Morgillo M., Vitelli N., Iannuzzi A., Aliberti E., Giallauria F., Tramontano A., Carluccio R. (2021). Lipoprotein(a) where do we stand? from the physiopathology to innovative terapy. Biomedicines.

[181] Rikhi R., Bhatia H.S., Schaich C.L., Ashburn N., Tsai M.Y., Michos E.D., Chevli P.A., Herrington D.M., Tsimikas S., Shapiro M.D. (2023). Association of Lp(a) (Lipoprotein[a]) and hypertension in primary prevention of cardiovascular disease: The MESA. Hypertension.

[182] Sechi L.A., Catena C., Casaccio D., Zingaro L. (2000). Lipoprotein(a), haemostatic variables and cardiovascular damage in hypertensive patients. J. Hypertens..

[183] Catena C., Novello M., Lapenna R., Baroselli S., Colussi G.L., Nadalini E., Favret G., Cavarape A., Soardo G., Sechi L.A. (2005). New risk factors for atherosclerosis in hypertension: Focus on the prothrombotic state and lipoprotein(a). J. Hypertens..

[184] Sechi L.A., Novello M., Colussi G.L., Di Fabio A., Chiuch A., Nadalini E., Casanova-Borca A., Uzzau A., Catena C. (2008). Relationship of plasma renin with a prothrombotic state in hypertension: Relevance for organ damage. Am. J. Hypertens..

[185] Mattace-Raso F.U.S., van der Cammen T.J.M., Hofman A., van Popele N.M., Bos M.L., Schalekamp M.A.D.H., Asmar R., Reneman R.S., Hoeks A.P.G., Bretler M.M.B. (2006). Arterial stiffness and risk of coronary heart disease and stroke: The Rotterdam Study. Circulation.

[186] Willum-Hansen T., Staessen J.A., Torp-Pedersen C., Rasmussen S., Thijs L., Ibsen H., Jeppesen J. (2006). Prognostic value of aortic pulse wave velocity as index of arterial stiffness in the general population. Circulation.

[187] Laurent S., Boutouyrie P., Asmar R., Gautier I., Laloux B., Guize L., Ducimetiere P., Benetos A. (2001). Aortic stiffness is an independent predictor of all-cause and cardiovascular mortality in hypertensive patients. Hypertension.

[188] Laurent S., Katsahian S., Fassot C., Tropeano A.I., Gautier I., Laloux B., Boutouyrie P. (2003). Aortic stiffness is an independent predictor of fatal stroke in essential hypertension. Stroke.

[189] Townsend R.R., Wilkinson I.B., Schiffrin E.L., Avolio A.P., Chirinos J.A., Cockroft J.R., Heffernan K.S., Lakatta E.G., McEniery C.M., Mitchell G.F. (2015). Recommendations for Improving and Standardizing Vascular Research on Arterial Stiffness: A Scientific Statement From the American Heart Association. Hypertension.

[190] Wildman R.P., Farhat G.N., Patel A.S., Mackey R.H., Brockwell S., Thompson T., Sutton-Tyrrell K. (2005). Weight change is associated with change in arterial stiffness among healthy young adults. Hypertension.

[191] Catena C., Colussi G., Frangipane A., Russo A., Verheyen N.D., Sechi L.A. (2015). Carotid artery stiffness is related to hyperinsulinemia and insulin-resistance in middle-aged, non-diabetic hypertensive patients. Nutr. Metab. Cardiovasc. Dis..

[192] Wilkinson I.B., Prasad K., Hall I.R., Thomas A., MacCallum H., Webb D.J., Frenneaux M.P., Cockroft J.R. (2002). Increased central pulse pressure and augmentation index in subjects with hypercholesterolemia. J. Am. Coll. Cardiol..

[193] Jatoi N.A., Jerrard-Dunne P., Feely J., Mahmud A. (2007). Impact of smoking and smoking cessation on arterial stiffness and aortic wave reflection in hypertension. Hypertension.

[194] Luft F.C. (2012). Molecular mechanisms of arterial stiffness: New insights. J. Am. Soc. Hypertens..

[195] Brosolo G., Da Porto A., Catena C., Sechi L.A. (2021). Arterial stiffening: Is it just high blood pressure. Rev. Cardiovasc. Med..

[196] Catena C., Colussi G.L., Brosolo G., Sechi L.A. (2012). A prothrombotic state is associated with early arterial damage in hypertensive patients. J. Atheroscler. Thromb..

[197] Brosolo G., Da Porto A., Bulfone L., Vacca A., Bertin N., Vivarelli C., Sechi L.A., Catena C. (2023). Association of arterial stiffness with a prothrombotic state in uncomplicated nondiabetic hypertensive patients. Front. Cardiovasc. Med..

[198] Wakabayashi I., Masuda H. (2006). Lipoprotein (a) as a determinant of arterial stiffness in elderly patients with type 2 diabetes mellitus. Clin. Chim. Acta.

[199] Morishita R., Ishii J., Kusumi Y., Yamada S., Komai N., Ohishi M., Nomura M., Hishida H., Niihashi M., Mitsumata M. (2009). Association of serum oxidized lipoprotein(a) concentration with coronary artery disease: Potential role of oxidized lipoprotein(a) in the vasucular wall. J. Atheroscl. Thromb..

[200] Kotani K., Yamada S., Yamada T., Kario K., Taniguchi N. (2013). Oxidized lipoprotein(a) and cardio-ankle vascular index (CAVI) in hypertensive subjects. Heart Vessel..

[201] Kronenberg F., Kathrein H., König P., Neyer U., Sturm W., Lhotta K., Gröchenig E., Utermann G., Dieplinger H. (1994). Apolipoprotein(a) phenotypes predict the risk for carotid atherosclerosis in patients with end-stage renal disease. Arterioscler. Thromb..

[202] Yun J.S., Ahn Y.B., Song K.H., Yoo K.D., Park Y.M., Kim H.W., Ko S.H. (2016). Lipoprotein(a) predicts a new onset of chronic kidney disease in people with Type 2 diabetes mellitus. Diabet. Med..

[203] Emdin C.A., Khera A.V., Natarajan P., Klarin D., Won H.H., Peloso G.M., Stitziel N.O., Nomura A., Zekavat S.M., Bick A.G. (2016). Phenotypic characterization of genetically lowered human lipoprotein(a) levels. J. Am. Coll. Cardiol..

[204] Kettunen J., Demirkan A., Wurtz P., Draisma H.H.M., Haller T., Rawal R., Vaarhorst A., Kangas A.J., Lyytikainen L.P., Pirinen M. (2016). Genome-wide study for circulating metabolites identifies 62 loci and reveals novel systemic effects of LPA. Nat. Commun..

[205] Rahman M., Yang W., Akkina S., Alper A., Anderson A.H., Appel L.J., He J., Raj D.S., Schelling J., Strauss L. (2014). Relation of serum lipids and lipoproteins with progression of CKD: The CRIC study. Clin. J. Am. Soc. Nephrol..

[206] Catena C., Colussi G.L., Nait F., Pezzutto F., Martinis F., Sechi L.A. (2015). Early renal failure as a cardiovascular disease: Focus on lipoprotein(a) and prothrombotic state. World J. Nephrol..

[207] Sechi L.A., Zingaro L., Bartoli E. (1999). Increased serum lipoprotein(a) levels in patients with renal failure. Ann. Intern. Med..

[208] Sechi L.A., Zingaro L., Catena C., Perin A., De Marchi S., Bartoli E. (1999). Lipoprotein(a) and apolipoprotein(a) isoforms and proteinuria in patients with moderate renal failure. Kidney Int..

[209] Catena C., Colussi G.L., Nait F., Capobianco F., Sechi L.A. (2015). Lipoprotein(a) levels and atherosclerotic renal artery stenosis in hypertensive patients. Kidney Blood Press. Res..

[210] Hornstra G., Van Houwelingen A.C., Kester A.D., Sundram K. (1991). A palm oil-enriched diet lowers serum lipoprotein (a) in normocholesterolemic volunteers. Atherosclerosis.

[211] Ginsberg H.N., Kris-Etherton P., Dennis B., Elmer P.J., Ershow A., Lefevre M., Pearson T., Roheim P., Ramakrishnan R., Reed R. (1998). Effects of reducing dietary saturated fatty acids on plasma lipids and lipoproteins in healthy subjects: The DELTA Study, protocol 1. Arterioscler. Thromb. Vasc. Biol..

[212] Berglund L., Lefevre M., Ginsberg H.N., Kris-Etherton P.M., Elmer P.J., Stewart P.W., Ershow A., Pearson T.A., Dennis B.H., Roheim P.S. (2007). Comparison of monounsaturated fat with carbohydrates as a replacement for saturated fat in subjects with a high metabolic risk profile: Studies in the fasting and postprandial states. Am. J. Clin. Nutr..

[213] Clevidence B.A., Judd J.T., Schaefer E.J., Jenner J.L., Lichtenstein A.H., Muesing R.A., Wittes J., Sunkin M.E. (1997). Plasma lipoprotein (a) levels in men and women consuming diets enriched in saturated, cis-, or trans-monounsaturated fatty acids. Arterioscler. Thromb. Vasc. Biol..

[214] Muller H., Lindman A.S., Blomfeldt A., Seljeflot I., Pedersen J.I. (2003). A diet rich in coconut oil reduces diurnal postprandial variations in circulating tissue plasminogen activator antigen and fasting lipoprotein (a) compared with a diet rich in unsaturated fat in women. J. Nutr..

[215] Müller H., Lindman A.S., Brantsæter A.L., Pedersen J.I. (2003). The serum LDL/HDL cholesterol ratio is influenced more favorably by exchanging saturated with unsaturated fat than by reducing saturated fat in the diet of women. J. Nutr..

[216] Faghihnia N., Tsimikas S., Miller E.R., Witztum J.L., Krauss R.M. (2010). Changes in lipoprotein(a), oxidized phospholipids, and LDL subclasses with a low-fat high-carbohydrate diet. J. Lipid Res..

[217] Berryman C.E., West S.G., Fleming J.A., Bordi P.L., Kris-Etherton P.M. (2015). Effects of daily almond consumption on cardiometabolic risk and abdominal adiposity in healthy adults with elevated LDL-cholesterol: A randomized controlled trial. J. Am. Heart Assoc..

[218] Jenkins D.J., Kendall C.W., Marchie A., Parker T.L., Connelly P.W., Qian W., Haight J.S., Faulkner D., Vidgen E., Lapsley K.G. (2002). Dose response of almonds on coronary heart disease risk factors: Blood lipids, oxidized low-density lipoproteins, lipoprotein (a), homocysteine, and pulmonary nitric oxide: A randomized, controlled, crossover trial. Circulation.

[219] Fraley A.E., Schwartz G.G., Olsson A.G., Kinlay S., Szarek M., Rifai N., Libby P., Ganz P., Witztum J.L., Tsimikas S. (2009). Relationship of oxidized phospholipids and biomarkers of oxidized low-density lipoprotein with cardiovascular risk factors, inflammatory biomarkers, and effect of statin therapy in patients with acute coronary syndromes: Results from the MIRACL (Myocardial Ischemia Reduction With Aggressive Cholesterol Lowering) trial. J. Am. Coll. Cardiol..

[220] Colussi G., Baroselli S., Sechi L.A. (2004). W-3 polyunsaturated fatty acids decrease plasma lipoprotein(a) levels in hypertensive subjects. Clin. Nutr..

[221] Croyal M., Ouguerram K., Passard M., Ferchaud-Roucher V., Chétiveaux M., Billon-Crossouard S., de Gouville A.C., Lambert G., Krempf M., Nobécourt E. (2015). Effects of extended-release nicotinic acid on apolipoprotein (a) kinetics in hypertriglyceridemic patients. Arterioscler. Thromb. Vasc. Biol..

[222] Lincoff A.M., Nicholls S.J., Riesmeyer J.S., Barter P.J., Brewer H.B., Fox K.A.A., Gibson C.M., Granger C., Menon V., Montalescot G. (2017). Evacetrapib and cardiovascular outcomes in high-risk vascular disease. N. Engl. J. Med..

[223] Thomas G.S., Cromwell W.C., Ali S., Chin W., Flaim J.D., Davidson M. (2013). Mipomersen, an apolipoprotein B synthesis inhibitor, reduces atherogenic lipoproteins in patients with severe hypercholesterolemia at high cardiovascular risk: A randomized, double-blind, placebo-controlled trial. J. Am. Coll. Cardiol..

[224] Sabatine M.S., Giugliano R.P., Keech A.C., Honarpour N., Wiviott S.D., Murphy S.A., Kuder J.F., Wang H., Liu T., Wasserman S.M. (2017). Evolocumab and clinical outcomes in patients with cardiovascular disease. N. Engl. J. Med..

[225] Bittner V.A., Szarek M., Aylward P.E., Bhatt D.L., Diaz R., Edelberg J.M., Fras Z., Goodman S.G., Halvorsen S., Hanotin C. (2020). Effect of alirocumab on lipoprotein(a) and cardiovascular risk after acute coronary syndrome. J. Am. Coll. Cardiol..

[226] Frank S., Gauster M., Strauss J., Hrzenjak A., Kostner G.M. (2001). Adenovirus-mediated apo(a)-antisense-RNA expression efficiently inhibits apo(a) synthesis in vitro and in vivo. Gene Ther..

[227] Tsimikas S., Karwatowska-Prokopczuk E., Gouni-Berthold I., Tardif J.C., Baum S.J., Elizabeth Steinhagen-Thiessen E., Shapiro M.D., Stroes E.S., Moriarty P.M., Nordestgaard B.G. (2020). Lipoprotein(a) reduction in persons with cardiovascular disease. N. Engl. J. Med..

[228] Koren M.J., Moriarty P.M., Baum S.J., Neutel J., Hernandez-Illas M., Weintraub H.S., Florio M., Kassahun H., Melquist S., Varrieur T. (2022). Preclinical development and phase 1 trial of a novel siRNA targeting lipoprotein(a). Nat. Med..

[229] Nissen S.E., Wolski K., Balog C., Swerdlow D.I., Scrimgeour A.C., Rambaran C., Wilson R.J., Boyce M., Ray K.K., Cho L. (2022). Single ascending dose study of a short interfering RNA targeting lipoprotein(a) production in individuals with elevated plasma lipoprotein(a) levels. JAMA.

